# Activated Carbon from Agricultural Wastes for Adsorption of Organic Pollutants

**DOI:** 10.3390/molecules25215105

**Published:** 2020-11-03

**Authors:** Magdalena Blachnio, Anna Derylo-Marczewska, Barbara Charmas, Malgorzata Zienkiewicz-Strzalka, Viktor Bogatyrov, Mariia Galaburda

**Affiliations:** 1 Faculty of Chemistry, Maria Curie-Sklodowska University, M. Curie-Sklodowska Sq. 3, 20-031 Lublin, Poland; barbara.charmas@poczta.umcs.lublin.pl (B.C.); malgorzata.zienkiewicz@poczta.umcs.lublin.pl (M.Z.-S.); 2 Chuiko Institute of Surface Chemistry of National Academy of Sciences of Ukraine, General Naumov Street 17, 03164 Kyiv, Ukraine; bogatyrov@isc.gov.ua (V.B.); mariia.galaburda@gmail.com (M.G.)

**Keywords:** activated carbon, dye adsorption, herbicide adsorption, adsorption kinetics, adsorption equilibrium

## Abstract

Agricultural waste materials (strawberry seeds and pistachio shells) were used for preparation of activated carbons by two various methods. Chemical activation using acetic acid and physical activation with gaseous agents (carbon dioxide and water vapor) were chosen as mild and environmentally friendly methods. The effect of type of raw material, temperature, and activation agent on the porous structure characteristics of the materials was discussed applying various methods of analysis. The best obtained activated carbons were characterized by high values of specific surface area (555–685 m^2^/g). The Guinier analysis of small-angle X-ray scattering (SAXS) curves showed that a time of activation affects pore size. The samples activated using carbon dioxide were characterized mostly by the spherical morphology of pores. Adsorbents were utilized for removal of the model organic pollutants from the single- and multicomponent systems. The adsorption capacities for the 4-chloro-2-methyphenoxyacetic acid (MCPA) removal were equal to 1.43–1.56 mmol/g; however, for adsorbent from strawberry seeds it was much lower. Slight effect of crystal violet presence on the MCPA adsorption and inversely was noticed as a result of adsorption in different types of pores. For similar herbicides strong competition in capacity and adsorption rate was observed. For analysis of kinetic data various equations were used.

## 1. Introduction

Development of many branches of industry, accompanied by increasingly stringent legislation for the environmental protection, results in a great need for cheap and effective adsorbents. Due to their availability and environmental neutrality as well as developed porosity, activated carbons have a wide range of applications. They are not only applied for the adsorption of pollutants from liquid or gas phase, but also in catalysis as catalysts, catalyst supports, or they serve to storage gas or desalinate media.

Currently the most popular activated carbons are produced from fossil coals and wood because of simplicity of developing porous structure during carbonization and activation using oxidizing gases. Commercial activated carbons are relatively expensive, thus more and more attempts are being made to use new raw materials and biomass. Due to high annual growth of cellulose biopolymers and other polysaccharides, the various waste materials seem to be an attractive precursor for the activated carbon production. They are cheap, ecological, renewable, and an easily accessible source of raw material and biomass. There are a wide variety of the waste materials used for production of effective activated carbons. In order to bring closer recent achievements related to the topic, some exemplary research reports may be presented. Özsin et al. obtained the activated carbons by chemical activation with KOH and K_2_CO_3_ and used them for removal of copper ions from aqueous solution [1]. In turn, Ghorbani et al. prepared activated the carbon from sugar beet bagasse for the chromium (VI) ions adsorption [2]. The research group of Ibrahim’ produced powdered carbon from *Leucaena leucocephala* biomass for the cadmium ions removal [3]. Borah et al. used activated carbon derived from tea waste for the eosin yellow dye adsorption [4]. Other researchers [5,6] studied decoloration of aqueous solutions from methylene blue and congo red on the activated carbons from rice straw and coffee waste, respectively. Cansado et al. recycled wood composites into activated carbon and used them for the removal of 4-chloro-2-methylphenoxy acetic acid (MCPA) from aqueous solutions [7]. Some researchers worked on formation of hybrid materials based on activated carbon and other components depending on the area of application. Ramamoorthy et al. carried out the synthesis of activated carbon from corn cob, then the product was enriched with SnO_2_ and used in the photodegradation of methylene blue under sunlight irradiation [8].

In Table 1 a comparison of the adsorption capacities for activated carbons from above-mentioned reports with alternative non-carbonaceous adsorbents is presented. The data indicate that adsorption effectiveness of activated carbons toward some pollutants (heavy metal ions, dyes, and herbicides) was mostly similar or higher than that of the other adsorbents. Only polyaniline/Fe_3_O_4_ [9] and sand/MgFe-layered double hydroxide composites [10] proved to be more efficient adsorbents. Obviously, depending on the type of adsorption system different interactions between pollutants and adsorbent were involved. The mechanisms based on physical forces (hydrogen bonding, Van der Waals force, and electrostatic force) and hydrophobic interactions for the organic compounds adsorption were proposed [6,7,10]. Meanwhile, the ion exchange and surface complexation mechanisms for the ion removal were indicated [2]. Regarding a performance of activated carbons as adsorbents, the adsorption methods proved to be simple and economical for the treatment of polluted waters. Although, the use of other conventional techniques such as photodegradation, oxidation with chemicals, filtration, coagulation, and sedimentation seem to be equally useful [8,11], some of them can generate harmful by-products or need to use of the environmentally burdening reagents [12].

The structure and properties of the activated carbon depend on the nature of precursor whose biochemical composition determines dynamics of the pyrolysis process, its yield, abrasion, and amount of ash [17,18]. Raw materials with a sufficiently high carbon content and low percentage of inorganics are preferable for the activated carbon production. The usage of agricultural raw materials and biomass contributes to sustainability of activated carbons production. Moreover, it reduces release of greenhouse gases because of lower contents of sulfur and nitrogen in comparison with fossil coals [19]. Due to abundant supply, high density, and purity, pistachio shells seem to be a suitable raw material for activated carbon production. Pistachio is widely cultivated in saline, dry, and hot areas of the Middle East, Mediterranean countries, and the United States [20]. Generally, the world production of pistachio has increased in recent years. As pistachio contains 18% of shell, approximately 100,000 tons of shells a year are obtained as a residue. Studies show that the fermentation and pyrolysis processes of pistachio shells can produce valuable biofuel. Production of bio-oil, biogas, and char was indicated simultaneously as an effective way to dispose pistachio wastes [21,22,23]. Another potential method of optimum utilization of pistachio shells is conversion to activated carbon. High carbon content and low percentage of inorganics make it a suitable precursor for this purpose. Reviewing literature on the subject, it turns out that there is a relatively small number of comparative studies which could develop knowledge about an impact of the activated carbon properties on effectiveness of organic pollutants adsorption from aqueous solutions in single- and multi-component systems taking into account both the sorption capacity and kinetics. For this reason, the investigations in this line are of both cognitive and practical importance enabling optimization of the adsorption methods for water and wastewater treatment technologies.

In our paper a series of activated carbons from agricultural wastes (mainly pistachio shells) was obtained in processes of chemical activation using acetic acid and physical activation with gaseous agents (carbon dioxide, water vapor). Differentiation of synthesis process conditions allowed obtaining adsorbents with a developed internal structure. Basing on the complex adsorption measurements (capacity and adsorption rate) the activated carbons effectiveness for the model organic pollutants removed from the one- and multicomponent systems was estimated. The physicochemical properties of carbons were studied by means of various techniques: adsorption/desorption of nitrogen, potentiometric titration, scanning electron microscope (SEM), transmission electron microscopy (TEM), X-ray photoelectron spectroscopy (XPS), small-angle X-ray scattering (SAXS), and thermal analysis. The model of adsorption on energetically heterogeneous solids was utilized for isotherm analysis (Generalized Langmuir isotherm equation (GL)). The measured concentration vs. time profiles for the adsorption systems were analyzed using the Elovich, pseudo-first-order (PFOE), pseudo-second-order (PSOE), mixed 1.2-order (MOE) equations as well as the multi-exponential equation (m-exp). The applicability of the kinetic equations was evaluated with regard to fitting quality and compatibility with the adsorption process mechanism.

## 2. Methods and Calculation Procedures

### 2.1. Preparation of Activated Carbons

Preparation of activated carbons was based on two various methods of activation: chemical using acetic acid and physical one with gaseous agents (carbon dioxide and water vapor). A chemical activation method for strawberry seeds precursor and a physical one for pistachio shells precursor were applied.

#### 2.1.1. Preparation of Activated Carbon Using Chemical Activation

The residue material after supercritical extraction of strawberry seeds as a powder of the size 0.20–0.70 mm was dried and subjected to chemical activation by acetic acid impregnation. After 24 h the sample was dried up to complete acid evaporation. Next, the sample was pyrolyzed at 600 °C at a heating rate of 10 °C/min in a quartz reactor heated by a vertical furnace with argon with a flow rate of 150 mL/min. The sample was kept at the final temperature for 2 h and then it was cooled in the inert atmosphere. The obtained activated carbon was denoted as Ac_Ar.

#### 2.1.2. Preparation of Activated Carbon Using Physical Activation

A series of activated carbons from pistachio shells was prepared by differentiation of the synthesis parameters such as activation time, atmosphere, or additional activation using a microwave reactor. The source material was used after submerging in water (ambient temperature) for two days to remove salt, then after drying were crushed to the size of 2–3 mm. The carbonization process was conducted at 400 °C (1 h) in a quartz reactor heated by a vertical furnace (heating rate: 10 °C/min) in the carbon dioxide (150 mL/min) atmosphere. Then, the char was subjected to physical activation at 800 °C in the carbon dioxide atmosphere for 1 h (the activated carbon is denoted as CD_1 h), and thereafter it was cooled in the inert atmosphere. The same preparation procedure was applied to the other activated carbons changing: the time of activation process (3 h)–the sample denoted CD_3 h; the time and activation atmosphere: simultaneous activation using carbon dioxide and water vapor (peristaltic pump, 0.6 mL/min) for 1 h–ST_CD_1 h; or 3 h–ST_CD_3 h. Part of the sample CD_1 h was additionally subjected to activation by water using the microwave source of energy (reactor NANO 2000, Plazmatronica, Wroclaw, Poland) in the pressure range with a power of 77–80 atm. for 1 h–CD_MV_1 h or 1.5 h–CD_MV_1.5 h.

#### 2.1.3. Schematic Description of the Adsorbents Preparation Procedures

- Ac_Ar-strawberry seeds (precursor), chemical activation (acetic acid), pyrolysis process (600 °C, 2 h, argon atmosphere);
- CD_1 h-pistachio shells (precursor), pyrolysis process (400 °C, 1 h, and carbon dioxide atmosphere), physical activation (800 °C, 1 h, carbon dioxide atmosphere);
- CD_3 h-pistachio shells (precursor), pyrolysis process (400 °C, 1 h, and carbon dioxide atmosphere), physical activation (800 °C, 3 h, carbon dioxide atmosphere);
- ST_CD_1 h-pistachio shells (precursor), pyrolysis process (400 °C, 1 h, carbon dioxide atmosphere), physical activation (800 °C, 1 h, carbon dioxide, and water vapor atmosphere);
- ST_CD_3 h-pistachio shells (precursor), pyrolysis process (400 °C, 1 h, carbon dioxide atmosphere), physical activation (800 °C, 3 h, carbon dioxide, and water vapor atmosphere);
- CD_MV_1 h-pistachio shells (precursor), pyrolysis process (400 °C, 1 h, carbon dioxide atmosphere), first stage of physical activation (800 °C, 1 h, and carbon dioxide atmosphere), second stage of physical activation (microwave radiation, 1 h);
- CD_MV_1.5 h-pistachio shells (precursor), pyrolysis process (400 °C, 1 h, and carbon dioxide atmosphere), first stage of physical activation (800 °C, 1 h, and carbon dioxide atmosphere), second stage of physical activation (microwave radiation, 1.5 h).

### 2.2. Adsorbates

For the present studies the commercial, chloride derivatives of phenoxyacids: MCPA (4-chloro-2-methyphenoxyacetic acid); 2.4-D (2.4-dichlorophenoxyacetic acid) and the derivative of triarylmethane: crystal violet (CV) of analytical or ACS reagent grade were purchased from Sigma–Aldrich, Acros and Alfa Aesar, respectively. The first two substances are widely used synthetic herbicides because of their low cost and good selectivity. Crystal violet is a basic dye with a relatively large molecular size. The chemical structures and the most relevant physicochemical properties of these organics are listed in Table 2.

### 2.3. Methods of Investigations

#### 2.3.1. Nitrogen Adsorption/Desorption Measurements

The porosity of the obtained adsorbents was analyzed using the low-temperature nitrogen adsorption/desorption isotherms measured at 77 K by the AUTOSORB-1CMS apparatus (Quantachrome Instruments, FL, USA). The values of the parameters characterizing the properties of porous structure were calculated: the BET specific surface area (*S_BET_*) (estimated from the linear BET plot of adsorption data), the total pore volume (*V_t_*) (from the adsorption value at the relative pressure *p/p*_o_~0.98), the external surface area (*S_ext_*), the micropore volume (*V_mic_*) (from the *t*-plot), and the pore size distributions (PSD) followed by the Barrett, Joyner, and Halenda (BJH) procedure and the Non-Local Density Functional Theory (NLDFT) approach (Micromeritics Instrument Corporation, Norcross, GA, USA).

#### 2.3.2. Small-Angle X-ray Scattering (SAXS)

The small-angle X-ray scattering (SAXS) measurements were performed using an Empyrean diffractometer (PANalytical, Malvern, United Kingdom ) equipped with CuKα radiation (λ = 1.5418 Å) applying transmission geometry in the range of 0.1 to 4 degrees of 2θ and EasySAXS software as a toolbox for SAXS data analysis. The relevant parameter to SAXS analysis is the momentum transfer or scattering vector *q* defined as $q=\frac{4\pi \cdot sin\theta }{\lambda }$ where *θ* is the angle between the incident beam and the detector measuring the scattered intensity, and λ is the wavelength of the radiation. Porod approximation was applied to treat the SAXS data for evaluation of interfacial area between the matrix and the scattering objects (pores). Here, the course of the scattering intensity at a higher q range plays a significant role. Porod constant (*k_p_*) can be applied for the determination of the specific surface area of the two-phase system using X-ray scattering at small-angle:

(1) $$\frac{S}{V}=\frac{4p\left(1-p\right)k_{p}}{Q_{p}}$$

where *p* and (1 − *p*) are the volumes of two phases, *k_p_* is the Porod’s constant, and *Q_p_* is the Porod’s invariant which is proportional to the mean-square density fluctuation of the whole scattering volume. Typically, when $\frac{S}{V}$ dependence is calculated by Dv(R) or Porod algorithm, the specific surface area from SAXS (S_SAXS_) can be calculated according to the equation

(2) $$S_{SAXS}=\frac{10000\cdot \frac{S}{V\;}\left[{Å}^{-1}\right]}{d\;\left[\frac{g}{cm^{3}}\right]}$$

where $\frac{S}{V}$ is the surface to volume ratio calculated from the distribution curve and *d* is the mass density of a material.

#### 2.3.3. Scanning Electron Microscopy/Transmission Electron Microscopy

Surface morphology of the activated carbons was studied by the field emission Scanning Electron Microscopy (SEM) employing a Quanta^TM^ 3D FEG (FEI Company, Washington County, OR, USA) apparatus operating at 30 kV.

The microstructure of the samples was analyzed by the Transmission Electron Microscopy (TEM) Titan G2 60–300 kV FEI Company. The tasted samples were grinded, poured over with ethanol, and homogenized by ultrasonication.

#### 2.3.4. Potentiometric Titration Measurements

The surface charge density and point of zero charge were determined from the potentiometric titration measurements of the acidified suspension of activated carbons using the base solution. The fixed ionic strength of each sorbent suspension, I = 0.1 mol/L, was used. The surface charge density of the solids was determined from the relation pH = f(V_NaOH_).

#### 2.3.5. X-ray Photoelectron Spectroscopy

The XPS spectra were recorded using the Multi-Chamber Analytical System (Prevac, Rogów, Poland) equipped with the monochromatic Kα-Al radiation (1486.6 eV) (Gammadata Scienta, Uppsala, Sweden) and an X-ray power of 450 W. Pressure in the analysis chamber was smaller than 8 × 10^−9^ Pa. The Casa XPS program was used for the XPS spectra processing and quantitative analysis of the solid surfaces.

#### 2.3.6. Adsorption Equilibrium

The adsorption isotherms for the aqueous solutions of organic compounds were measured using the static method. For this purpose, the stock solutions were prepared dissolving one or two organics in the redistilled water: MCPA, 2,4-D, CV, CV + MCPA (1:20 relative molar concentration), and MCPA + 2.4-D (equimolar concentration). The initial solution concentrations of 0.08–3.4 mmol/L (MCPA); 0.08–2.2 mmol/L (2.4-D), 0.0078–0.098 mmol/L (CV), 0.0078–0.098 mmol/L (CV plus MCPA with higher concentrations, respectively), and 0.04–1.8 mmol/L (MCPA and 2.4-D, for each substance) were prepared from the stock solutions. Then, 40 mg samples of adsorbents were added to the flasks with 50 cm^3^ of organics solutions and shaken for 7 days at 25 °C at a speed of 110 rpm.

For the single and binary systems of CV and MCPA, the equilibrium solute concentrations were calculated applying spectrophotometric measurements (Cary 4000, Varian Inc., Victoria, Australia) at λ = 582 nm (CV) and λ = 278 nm (MCPA). The adsorbed amounts of substances were calculated from the mass balance equation.

The concentrations of binary solutions were based on the Beer–Walter’s Law and the additivity law expressing the total absorbance of the system as the sum of the absorbance of individual components. The condition of its compliance by the system is a lack of interactions between the components absorbing UV–Vis light.

The equilibrium solute concentrations of the binary system MCPA and 2.4-D were determined using a high-performance liquid chromatograph (HPLC) with the DAD detector (Shimadzu, Kyoto, Japan) The chromatographic analysis was conducted under the isocratic conditions on a Kinetex 5u C18 100A 150 × 4.6 mm column (Phenomenex, CA, USA) thermostated at 25 °C. The mobile phase was composed of acetonitrile and citrate phosphate buffer of pH~7.4 (80/20, *v*/*v*). The flow rate was fixed to 1 cm^3^/min and the absorption data measured at λ = 284 nm (2.4-D) and λ = 278 nm (MCPA). The same procedure for the single solute concentrations of herbicides was applied.

Using the results obtained from both analytical methods the uptake of adsorbates at equilibrium was calculated from the mass balance equation.

The experimental adsorption isotherms were analyzed using the Generalized Langmuir (GL) isotherm equation [24,25] based on the general theory of adsorption:

(3) $$\theta ={\left[\frac{{\left(Kc_{eq}\right)}^{n}}{1+{\left(Kc_{eq}\right)}^{n}}\right]}^{m/n}$$

where *θ = a_eq_/a_m_* is the global (overall) adsorption isotherm, *a_eq_* is the equilibrium adsorbed amount (mmol/g), *a_m_* is the adsorption capacity, *c_eq_* is the equilibrium solute concentration (mmol/L), *m* and *n* are the heterogeneity parameters characterizing a shape (width and asymmetry) of adsorption energy distribution function, where 0 < *m,n* ≤ 1 (*m* < *n* corresponds to the energy distribution extended towards high energies, and *m > n*-towards low energies, *m* = *n* = 1 corresponds to the energetically homogeneous system); *K* is the equilibrium constant characterizing a position of distribution function on the energy axis. For specific values of heterogeneity parameters, the GL equation reduces to simpler isotherms:

Langmuir–Freundlich (LF) (GL: 0 < *m = n* ≤ 1):

(4) $$\theta =\frac{{\left(Kc_{eq}\right)}^{m}}{1+{\left(Kc_{eq}\right)}^{m}}$$

Generalized Freundlich (GF) (GL: *n* = 1, 0 < *m* ≤ 1):

(5) $$\theta ={\left[\frac{Kc_{eq}}{1+Kc_{eq}}\right]}^{m}$$

Tóth (T) (GL: *m* = 1, 0 < *n* ≤ 1):

(6) $$\theta =\frac{Kc_{eq}}{{\left[1+{\left(Kc_{eq}\right)}^{n}\right]}^{1/n}}$$

Langmuir (L) (GL: *m = n* = 1):

(7) $$\theta =\frac{Kc_{eq}}{1+Kc_{eq}}$$

#### 2.3.7. Adsorption Kinetics

Kinetic measurements of single (CV), (MCPA) and binary (CV plus MCPA) systems were performed applying the UV–Vis spectrophotometer Cary 100 (Varian Inc., Victoria, Australia) with a flow cell working in a closed loop. The initial solute concentrations of single and binary solutions (with the 1:20 relative molar concentration ratio) were 0.75 mmol/L and 0.037 mmol/L for MCPA and CV, respectively (the range of absorbance linearity); 50 cm^3^ of solution was contacted with 100 mg of adsorbent in a thermostatic vessel (25 °C) and stirred mechanically during the experiment (110 rpm). The concentration vs. time profiles for the experimental systems were calculated from the recorded spectra.

For the binary system (MCPA plus 2.4-D) the kinetic measurements were performed by means of the chromatographic method. The 500 mg sample of adsorbent was added to the solution of the initial concentration 0.75 mmol/L of both herbicides (volume 250 cm^3^) in the thermostatic vessel (25 °C) with a mechanical stirrer (110 rpm). The solution samples (200 µL) were collected to vials and measured in accordance with the previously described procedure. The applied method allowed to determine precisely the adsorbate concentrations in binary solutions for the substances belonging to the same subgroup of organics (halogenated phenoxyacids) with the similar chemical structure (type of functional groups at the aromatic ring is various). Due to the overlap of herbicides absorption spectra application of the spectrophotometric method is unfeasible. For comparison, the same experimental conditions for single-solute systems were applied.

The kinetic profiles were analyzed using several equations commonly applied in the literature: Elovich, Lagergren (PFOE), SOE/PSOE, the mixed 1.2-order (MOE), and the multi-exponential (m-exp) equations.

The first- and second-order kinetic equations can be treated as phenomenological solutions describing processes driven by various mechanisms. Both equations can be written as a function dependent on concentration (*n*-th order rate equations) or as a function dependent on adsorption (pseudo-n-th order rate equations) [26,27,28,29,30]:

(8) $$\frac{dF}{dt}=k_{n}{\left(1-F\right)}^{n}$$

where *t* is the time, $F=\frac{a-a_{o}}{a_{eq}-a_{o}}$ or $F=\frac{\left(\theta -\theta_{o}\right)}{\left(\theta_{eq}-\theta_{o}\right)}$ and $F=\frac{c_{o}-c}{\left(c_{o}-c_{eq}\right)}$ is the adsorption progress, the subscript “o” means the initial value (here it is assumed: *a_o_* = 0, *θ_o_* = 0), *k_n_* is the rate coefficient.

The other forms of the concentration and adsorption rate equations are as follows,

(9) $$\frac{dc}{dt}=-k_{nc}{\left(c-c_{eq}\right)}^{n}\;\mathrm{and}\;\frac{da}{dt}=k_{na}{\left(a_{eq}-a\right)}^{n}$$

where $k_{n}=k_{n}a_{eq}^{n-1}$, $k_{n}=k_{nc}{\left(c_{o}-c_{eq}\right)}^{n-1}$, with $k_{1}=k_{1c}=k_{1a}$ and $k_{2}=k_{2c}\left(c_{o}-c_{eq}\right)=k_{2a}a_{eq}$ or in the integrated form for the order *n* = 1:

(10) $$F=1-\exp\left(-k_{1}t\right)\;\mathrm{and}\;\ln\left(1-F\right)=-k_{1}t$$

(11) $$\ln\left(\;c-c_{eq}\right)=\ln\left(c_{o}-c_{eq}\right)-k_{1}t$$

(12) $$\ln\left(\;a_{eq}-a\right)=\ln a_{eq}-k_{1}t$$

If *n* ≥ 2 the equation is as follows [31,32],

(13) $$F=\sqrt[{n-1}]{\frac{k_{n}^{’}t}{1+k_{n}^{’}t}}\;\;\mathrm{or}\;F^{n-1}=\frac{k_{n}^{’}t}{1+k_{n}^{’}t}$$

where $k_{n}^{’}=\left(n-1\right)k_{n}$.

If *n* = 2 it has a simpler form:

(14) $$F=\frac{k_{2}t}{1+k_{2}t}$$

The pseudo-second-order equation (PSOE) is often used as the linear dependence t/a vs. t (where $k_{2}=k_{2a}a_{eq}$) [31,33,34]:

(15) $$\frac{t}{a}=\frac{1}{k_{2a}a_{eq}}+\frac{t}{a_{eq}}$$

To highlight the deviations from the SOE/PSOE equation, PSOE as an alternative linear plot can be used [26,27,31,32,33,34]:

(16) $$a=a_{eq}-\frac{1}{k_{2}}\;\frac{a}{t}$$

A relatively simple analytical solution can be obtained applying the mixed 1.2-order equation (MOE) that is a combination of 1st and 2nd order processes for both concentration and adsorption rate dependences. This empirical equation corresponds to the intermediate behavior of the experimental system, between PFOE and PSOE, as a model generalization of these equations [26,33,34]:

(17) $$F=\frac{1-\exp\left(-k_{1}t\right)\;}{1-f_{2}\exp\left(-k_{1}t\right)}\;\;\mathrm{and}\;\ln\left(\frac{1-F}{1-f_{2}F}\right)=-k_{1}t$$

where *f_2_ < 1* is the normalized share of second-order term in the overall rate dependence.

MOE has close relation with the hybrid-order rate equation [35] and can be treated as a mathematical equivalent to the integrated Langmuir kinetic equation.

In the systems in which the equilibrium state has not been achieved yet and the chemical adsorption is assumed to occur, the Elovich equation can be of use. Some authors e.g., Riahi et al. and Wu et al. [36,37] used this equation to describe the middle part of diffusion kinetics.

(18) $$\frac{da}{dt}=\alpha \exp\left(-\beta a\right)$$

Integrated form (initially: *a* = 0, *t* = 0):

(19) $$a=\frac{1}{\beta }\left[\ln\left(\alpha \beta \right)+\ln\left(t+\frac{1}{\alpha \beta }\right)\right]$$

(20) $$c=c_{0}-\frac{V}{w\beta }[\ln\left(\alpha \beta \right)+\ln(t+t_{0})\;$$

(21) $$c=c_{0}-A-B\ln\left(t+t_{0}\right)$$

where *a* is the amount of adsorption at time *t*, *α* is the initial adsorption rate, *β* is a constant, *V* is the volume, *w* is the mass of adsorbent, and *A* and *B* are parameters.

Good results in the analysis of various physicochemical and adsorption processes can be obtained using the multi-exponential equation (m-exp). In the latter process it may be considered as a rough estimation of parallel series of the first-order processes (in structurally or energetically differentiated adsorption systems) or a series of follow up processes (e.g., inside the porous system with pore constrictions).

Generally, the multi-exponential equation (m-exp) can be written [26,27,31,33,34,38,39,40,41] as

(22) $$F=1-\sum_{i=1}^{n}f_{i}\exp\left(-k_{i}t\right)\;\mathrm{where}\;\sum_{i=1}^{n}f_{i}=1$$

(23) $$c=\left(c_{o}-c_{eq}\right)\;\overset{n}{\underset{i=1}{\sum }}f_{i}\exp\left(-k_{i}t\right)+\;c_{eq}$$

(24) $$a=a_{eq}\left[1-\overset{n}{\underset{i=1}{\sum }}f_{i}\exp\left(-k_{i}t\right)\right]$$

where *n* is the number of exponential terms, the coefficient *f_i_* (*i* = 1,2 … *n*) indicates the fraction of adsorbed equilibrium amount *a_eq_* ($f_{i}=\frac{a_{i}}{a_{eq}}$) corresponding to the adsorption process characterized by the rate coefficient *k_i_*.

The multi-exponential equation characterizes well enough the experimental systems with the fast initial adsorption process followed by the slower ones. Such phenomenon often occurs in the porous adsorbents with a nonuniform pore structure or a complex pore system with differentiated accessibility of smaller and larger pores. In practice the multi-exponential equation usually gives better optimization of kinetic data than any of the above-mentioned simple equations.

#### 2.3.8. Thermal Analysis

Thermal analysis of activated carbons was conducted using a STA 449 Jupiter F1 instrument, (Netzsch, Selb, Germany). The measurements in synthetic air atmosphere (50 cm^3^/min) at a temperature range of 30 to 1200 °C (heating rate 10 °C/min) were carried out. The standard Al_2_O_3_ crucible and thermocouple of type S as TG-DSC sensor were applied. The gaseous products released during decomposition of samples were analyzed by FTIR spectrometer (Bruker, Ettlingen, Germany) and by quadrupole MS spectrometer QMS 403C Aeölos (Netzsch, Selb, Germany) coupled on-line to STA instrument.

## 3. Results and Discussion

### 3.1. Characterization of the Adsorbents

**Figure 1:**
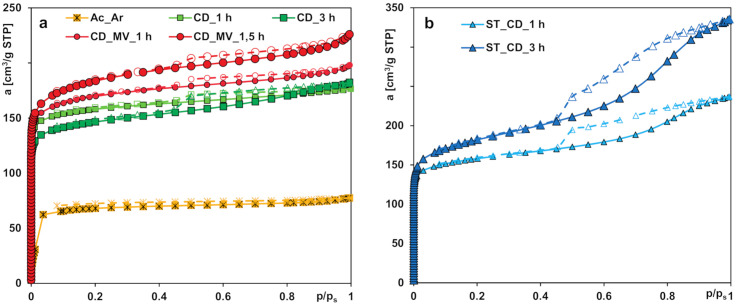
The nitrogen adsorption/desorption isotherms for the activated carbons (**a**) prepared by chemical activation (Ac_Ar) and physical activation using carbon dioxide (other samples), (**b**) prepared by physical activation using carbon dioxide and water vapor.

The nitrogen adsorption/desorption isotherms for the activated carbons prepared from two agricultural waste precursors under different conditions of the synthesis process are shown in Figure 1a,b. According to the IUPAC classification, one can find that the isotherms are hybrids of type I (in the range of low relative pressures; sharp increase of adsorption for *p*/*p_s_* < 0.05 is typical of microporous solids) and type IV (in the range of moderate and high pressures; formation of hysteresis loops).

In general, the adsorption isotherms exhibit a steep increase in the gas uptake at low relative pressures, corresponding to the micropores filling; thus, microporosity in the porous structures was developed during the synthesis process. However, differentiated amounts of gas uptake and different shapes of hysteresis loops at moderate and high relative pressures indicate the development of various extents of mesoporosity. In the case of the samples ST_CD_1 h and ST_CD_3 h, a significant increase in nitrogen adsorption at moderate and high relative pressures (with a higher slope) is observed, which indicates the increase in mesoporosity due to the pore widening and partial pore collapse. The hysteresis loops for the adsorbents belong to type H4, suggesting existence of the slit shaped mesopores. The carbon Ac_Ar is characterized by the slightest development of porous structure.

The differences the isotherms shape and nitrogen uptake reflect an essential variation of porous structure parameters summarized in Table 3. The lowest specific surface area (243 m^2^/g) as well as the total and micropore volumes (0.12 and 0.07 cm^3^/g, respectively) characterize the activated carbon Ac_Ar obtained from the residue material after the supercritical extraction of strawberry seeds. The sample preparation consisted in chemical activation by acetic acid impregnation and pyrolysis at 600 °C in the argon atmosphere for 2 h. The biochemical composition of precursor (relatively high percentage of inorganics) is supposed to affect poor structural parameters and a significant amount of ash in the final product. This assumption is confirmed by a significant residual mass of raw precursor after the thermogravimetric analysis (TGA) in the synthetic air atmosphere (up to 3.5% while for the pistachio shell it was only 1%).

A series of activated carbons obtained from the pistachio shells as a precursor is characterized by well-developed porosity with differentiated contribution of micro- and mesopores. Generally, all these samples were subjected to pyrolysis in the same manner, however, various activation time, different gaseous agents or additional activation by water vapor using microwave energy were applied. After activation with carbon dioxide for 1 h the sample CD_1 h shows the greatest contribution of micropores to the total pore volume (74%) and a relatively high specific surface area (599 m^2^/g). Extension of the activation time up to 3 h results in widening the existing micropores that is reflected in the increase in *S_ext_*, *V_t_* and the decrease in *S_BET_*, *V_mic_*. Moreover, the value of the median micropore width *D_mo_* calculated using the Horvath–Kawazoe method also increases slightly with the increasing activation time.

Simultaneous application of carbon dioxide and water vapor as activation agents (ST_CD_1 h) affects development of both micro- and mesoporosity compared to a method that uses merely carbon dioxide (CD_1 h). A significant increase in *V_t_* and *S_ext_* which is even much more visible for the sample treated with gases for 3 h (ST_CD_3 h) is noteworthy. Then, a substantial increase in the specific surface area (669 m^2^/g) is observed despite a small contribution of micropores to the total pore volume (32%). A significant increase in the median pore width *D_mo_* is also visible.

During the additional activation of the sample CD_1 h by means of water vapor heated by microwave radiarion the increase of *S_BET_, S_ext_*, and *V_t_* related to subsequent creation of pores and widening of the existing ones (decrease of micropores contribution to the total pore volume from 74 to 63%) is observed. However, extending the time of exposure to the microwaves from 1 to 1,5 h (samples CD_MV_1 h and CD_MV_1,5 h, respectively) results in development of microporosity (increase of *S_BET_, V_t_, V_mic_*, by over 68% of micropores in the total adsorption volume).

**Figure 2:**
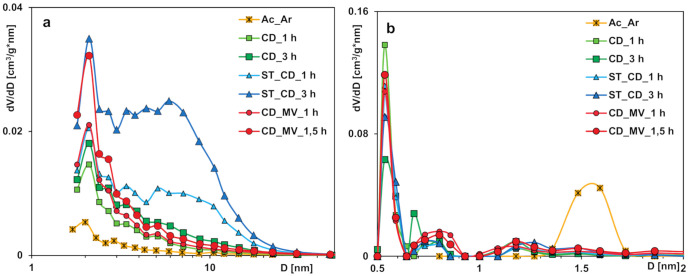
Pore size distributions calculated using the Barrett, Joyner, and Halenda BJH (**a**) and Non-Local Density Functional Theory NLDFT (**b**) methods.

The analysis of pore size distributions (Figure 2) calculated from the adsorption branch (reflecting inner pores diameter), the micropore size distributions (from the NLDFT method) and the median pore width *D_mo_* confirms the structural changes of the studied activated carbons. In general, preparation conditions significantly influence the characteristics of activated carbons; the obtained materials are predominantly microporous with great contribution of pores in the sub-micropore and supermicropore ranges. However, in the case of two samples prepared with simultaneous application of carbon dioxide and overheated water vapor as activation agents, materials with predominantly mesoporous structure but well-developed specific surface area were obtained. This characteristic of adsorbents is particularly advantageous from the point of view of the environmental protection because of enabling effective adsorption of both small and large molecules of organic pollutants in the multicomponent systems.

**Figure 3:**
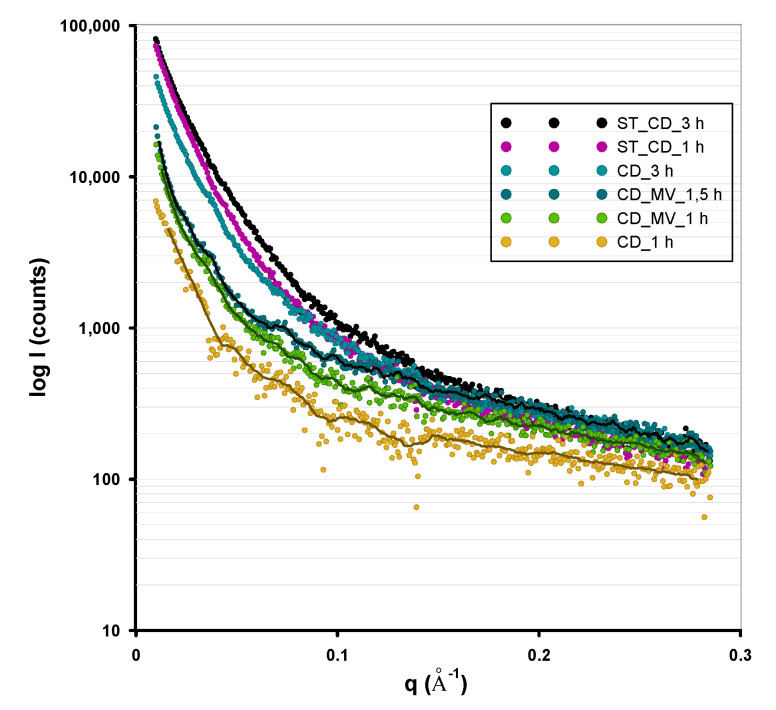
Scattering data as small-angle X-ray scattering (SAXS) profiles for activated carbon samples.

In the analysis of inhomogeneous structures at the nanoscale, the method based on the small-angle X-ray scattering seems to be extremely valuable. The SAXS data and general shape of the small-scale scattering curve depends mainly on the shape and size of nanoobjects and is not dependent on the atomic structure of the investigated materials. Thus, the SAXS analysis of the activated carbons can be used to detect and characterize the nanovoids of the porous system. According to the theory, the SAXS intensity should increase with the number of nanoobjects in the matrix. Figure 3 shows the dependence of the intensity logarithm, log*I*, on the scattering vector, *q*. From the SAXS curves, the significant differences in the scattering intensity are observed. This can be the first signal, that some changes in porous structure take place as a result various conditions in the activation step. In all of the investigated series of the samples (activation time as a variable parameter) the scattering intensity is greater when the activation time increase. A detailed investigation of the Guinier plots for all the samples reveals the linear regions in the low *q* regions characterized by a narrow *q* range and relatively large size of nanoobject, followed by wide *q* range in the higher *q* region describing smaller particles.

**Figure 4:**
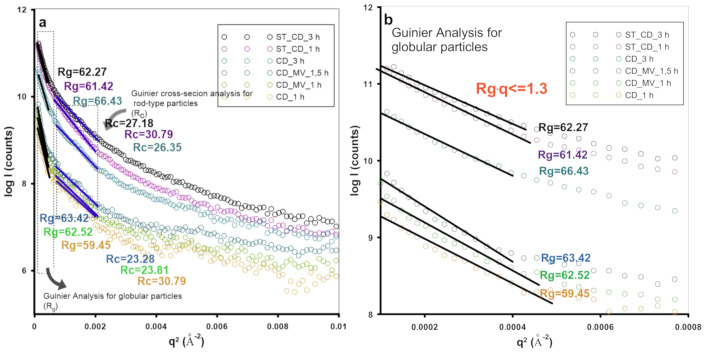

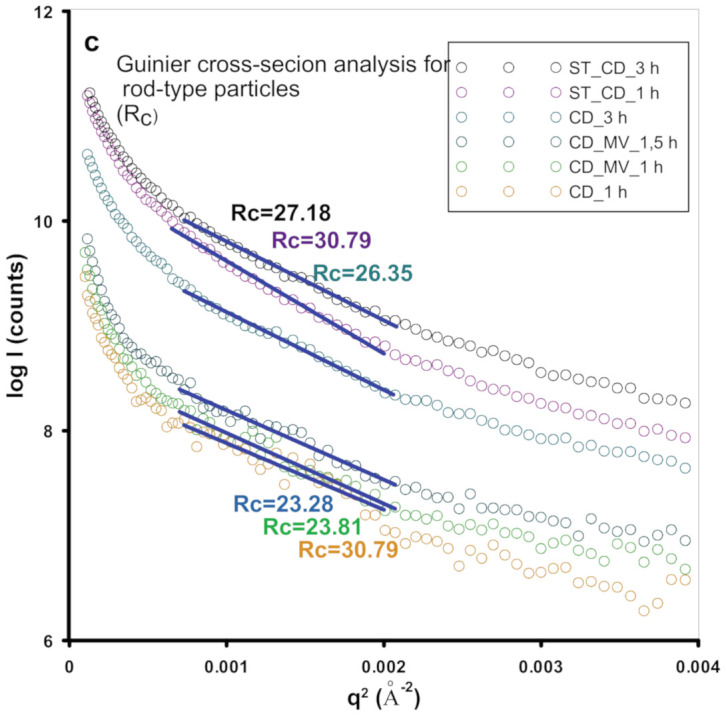
Guinier plots showing linear regions for two types of particles with calculation of gyration radius Rg and gyration radius of cross section R_c_ (**a**) corresponding Guinier analysis for globular particles (**b**) and rod-type particles (**c**). The estimated aggregate radius R_g_ is valid in the q range determined by qR < 1.3. The estimated aggregate radius of cross section R_c_ was determined by 1 < qR_c_ < 1.3.

Figure 4 shows the Guinier analysis of SAXS curves for various shape of inhomogeneities. The SAXS curve is described by A. Guinier’s approximation when the relation 0.1 < *qRg* < 1 is fulfilled (*Rg* is defined as radius of gyration of the particles). A nonlinear dependence of log(*I*) = f(*q*^2^) indicates the presence of agglomeration and morphological disorders compared to the model assumptions. In the case of investigated carbon samples, the linear range of the Guinier curves for particles with spherical morphology is quite narrow, what may suggest the small amount of this type of inhomogeneities in the whole matrix. However, this type of inhomogeneity (porosity) was confirmed for all samples of carbonaceous materials. The radius of gyration *Rg* which is the parameter describing the scattering ability and compared with the diameter of carbon particles is similar for all investigated samples, and varies from 59.49Å to 66.40Å, and suggests the size of spherical pores around 6 nm. The largest differences in the size of the scattering particles with spherical morphology were observed for CD_1 h and CD_3 h being one of the pairs that differ only in the time of activation—59.49Å and 66.40Å, respectively. This suggests the size increase of the particles responsible for X-rays scattering at such small angles (increasing the pore size) according to the activation time. A similar effect was observed for other pairs of samples. Generally, the increasing time of activation generates the increasing pore size, and moreover, the highest efficiency in the development of porous structures with spherical morphology was noted for the activation with carbon dioxide. Additionally, the Guinier analysis for rod-type particles was also performed due to the significant linear region visible in higher *q* values. In this case, the linear region was significantly wider. The radius of gyration for rod-type particles (*R_c_*) suggest the size of pores ~2–3 nm, and the size decreases with the increasing time of activation. This may suggest the creation of new pores during activation and their gradual increase in the process. Moreover, when the greater deviations from Guinier’s approximation are observed, the more non-dimensional and elongated form is present in the sample. This observation was noticed for greater values of the *q* parameter. This is the case for smaller objects, i.e., scattering in the range of higher *q* values. The results of SAXS analysis should be compared with the analysis of pore size distributions from low-temperature N_2_ adsorption/desorption. The comparison of the scattering intensity (*I*(*q*)) with the *S_BET_* values is justified. In both cases, the *S_BET_* and *I*(*q*) values increase when the longer time of activation was applied (one exception is in the case of CD-1 h and CD_3 h samples where the *S_BET_* doesn’t change so directly). In both cases, the good correlation was also obtained in the size of pores determined by SAXS and HK pore size distributions. Especially for rod-type structures, their size was surprisingly similar (from 2.3 to 3.1 nm from SAXS and 2–3 nm from HK).

**Figure 5:**
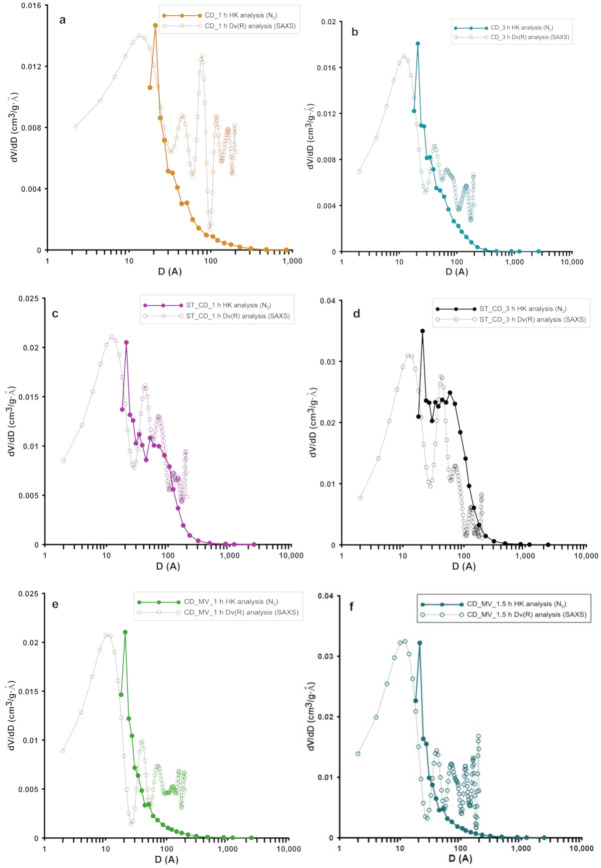
Calculated volume weighted particle size distributions of the particles from SAXS and comparison with distribution curves determined by HK (N_2_ adsorption/desorption) for all investigated samples (**a–f**), (**a**) CD _1 h, (**b**) CD_3 h, (**c**) ST_CD_1 h, (**d**) ST_CD_3 h, (**e**) CD_MV_1 h and **f**) CD_MV_1.5 h.

Moreover, the pore size distribution from SAXS data was also calculated and compared with the one obtained from nitrogen adsorption/desorption isotherms. Figure 5 shows the experimental HK and calculated SAXS distributions. In all cases the obtained results are very similar, although the data were registered with two such different techniques. A distinctive feature is the slight displacement of the maxima of particle size from the SAXS method towards slightly smaller dimensions (~1nm). However, the SAXS curves seem to be slightly more precise and sensitive to changes in the particle size. Recall that the basis of the SAXS technique is the difference in the electron density of the scattering media, and this contrast may be not so pronounced for the nitrogen molecules in the N_2_ method. Furthermore, the low-temperature N_2_ adsorption/desorption technique allows testing only the open porosity available for nitrogen (or other gas) penetration. The effect of small-angle scattering is suitable for both open and closed porosity. The differences between both techniques may describe the characteristics of closed porosity.

**Figure 6:**
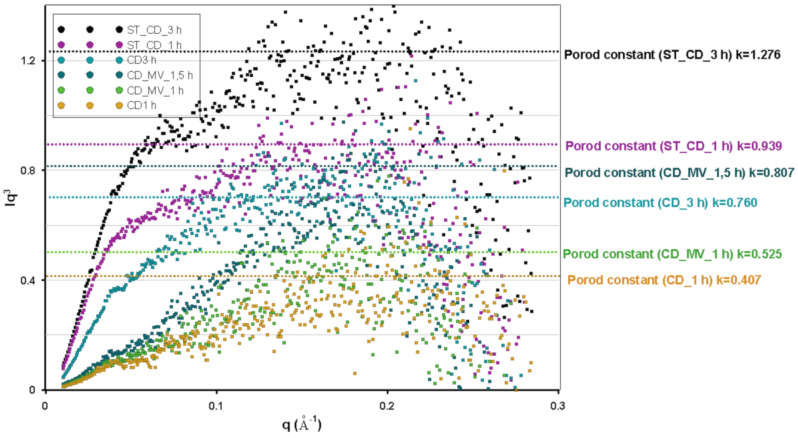
Porod plots of investigated carbons. The approximation and values of Porod constant values.

Porod’s law is one of the basic theoretical interpretations of SAXS data [42,43]. Figure 6 shows the plots of the Porod approximation of experimental scattering data where an asymptotic course in the high range of scattering vector was confirmed for the investigated carbons. The asymptotic behavior of the SAXS function was confirmed by the well-defined Porod constant values (Table 4). The linear course of Porod function indicates the presence of a separated interface between phases with various electron densities. Because the higher q values correspond to the smaller interparticle distance, surface area corresponding to the internal structure around the particle external layers is dominated. The level of linearity of the Porod range is various for the tested carbons. It means that the specific surface area varies depending on the applied procedure of their preparation and activation steps. General trends of these changes comply with the data obtained from low-temperature nitrogen sorption measurements. Especially, the activation procedure with carbon dioxide for 1 h (sample marked as (CD_1 h)) leads to receive a material with higher surface area than for sample (CD_3 h) activated by 3 hrs. Here, S_SAXS_ was defined as 592 m^2^/g and 620 m^2^/g for CD_1 and CD_3 h samples, respectively. Next, the application of carbon dioxide and water vapor as activation agents for ST_CD_1 h and ST_CD_3 h samples was responsible for obtaining the carbons of satisfactory specific surface area equal to 540 and 550 m^2^/g, respectively. Finally, the slightly higher values (743 m^2^/g and 771 m^2^/g) for CD_MV_1 h and CD_MV_1.5 h were obtained by this method in comparison to those calculated from the nitrogen adsorption data. Slightly higher values of surface areas calculated from the SAXS data, compared to the sorption method may be related to the presence of closed porosity, inaccessible to nitrogen molecules. Such observation leads to the assumption that the transitional stage of unfolding the porous structure during activation by microwaves has been revealed.

**Figure 7:**
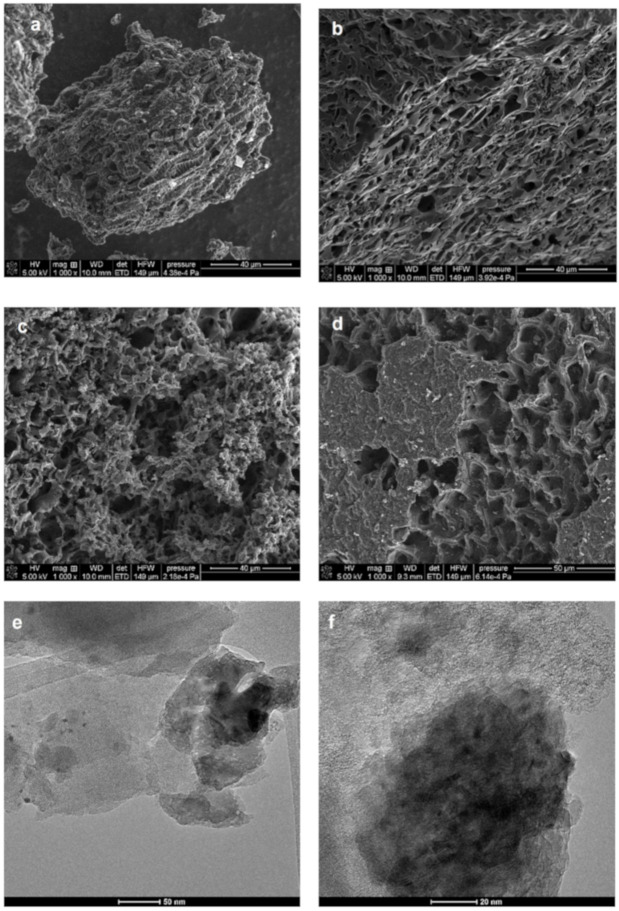

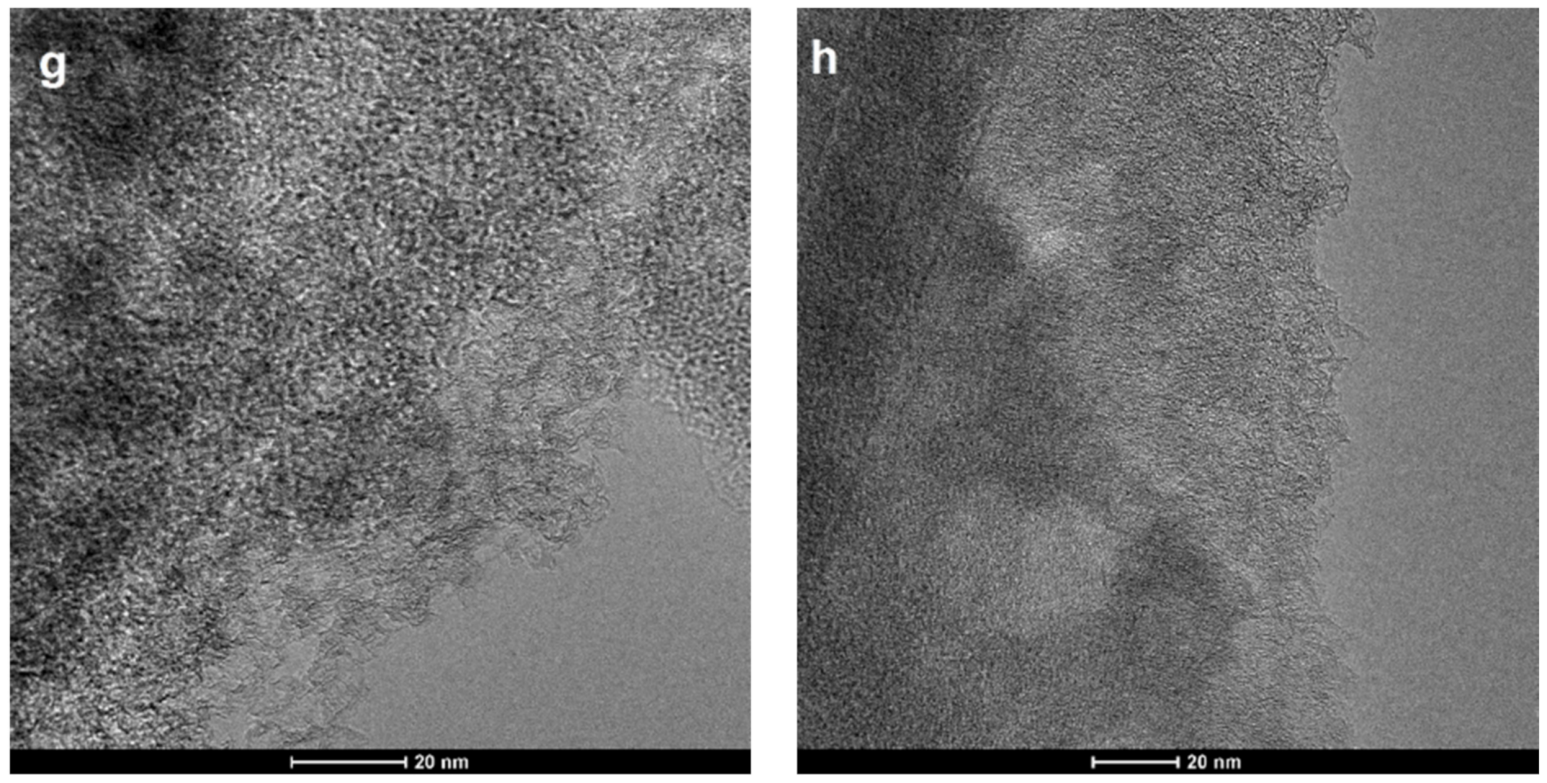
The SEM and TEM images of the following samples; Ac_Ar (**a**,**e**), CD_3 h (**b**,**f**), ST_CD_3 h (**c**,**g**), and CD_MV_1.5 h (**d**,**h**).

In Figure 7, the SEM images of the chosen activated carbons at 1000 magnifications are presented. The grain size of the sample Ac_Ar is lower than that of the others; however, the surface morphology of all solids shows roughness and irregularity. The most heterogeneous morphology of surface seems to occur for the activated carbons after activation by water vapor that results from the applied strong oxidation. The TEM microphotographs indicate the disordered and curled single carbon layers which consist of random cross-linked graphite-like planes.

**Figure 8:**
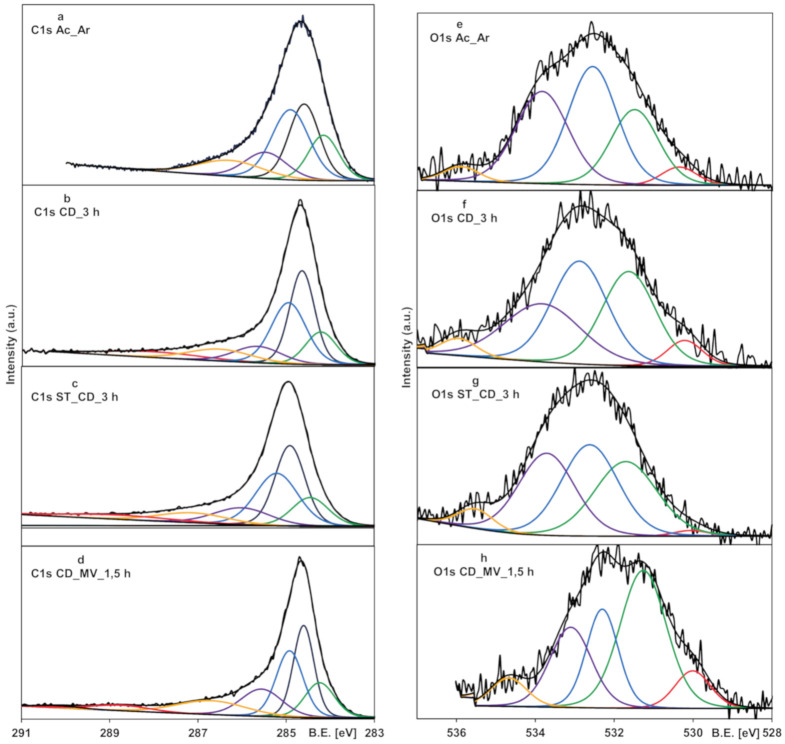
The fitted C 1s (**a–d**) and O 1s (**e**–**h**) XPS spectra for activated carbons.

The surface chemical composition of the chosen activated carbons from the XPS survey scans and the contribution of carbon and oxygen species estimated from the deconvolution of peaks to carbon (C1s) and oxygen (O1s) are presented in Table 5. The chemical composition of the sample Ac_Ar is as follows; carbon (86.8%), oxygen (9.5%), nitrogen (2.5%), phosphorus (0.8%), and silicon (0.49%). For the other samples, only carbon (94.1–94.7%) and oxygen (5.4–5.9%) were found. Undoubtedly, these differences are due to chemical composition of the precursors (strawberry seeds rich in minerals compared to the cellulose pistachio shells) and the preparation methods (higher temperature favors removal of non-carbon elements in the gaseous form). Generally, in the series of activated carbons obtained from the pistachio shells, the atomic content of oxygen is comparable and relatively low, which is characteristic of the adsorbents activated at high temperature in the gaseous atmosphere [44]. Deconvolution of the C1s spectra (Figure 8, left side) yields several peaks with the binding energies representing carbon as C=C sp^2^ (284.2–284.3 eV), C-C or C-H sp^3^ (284.6–284.7 eV) [45], C-C high sp^3^ in small clusters containing C=O bonds (284.9–285 eV) and the other forms of carbon bound to oxygen such as hydroxy- and ether-like groups (285.5–285.7 eV), carbonyl or quinone groups (286.3–286.7 eV), carboxyl or ester groups (anhydride) (288–288.8 eV), carbonate groups (290.2 eV) [46,47]. The O1s spectra for the activated carbons (Figure 8, right side) display five peaks corresponding to metal oxides (529.9–530.4 eV), carbonyl, lactone or carboxyl groups (531.3–532 eV), ether or hydroxyl groups bound to aliphatics (532.3–533 eV), ether or hydroxyl groups bound to aromatics (533.1–533.8 eV), adsorbed water or oxygen (534.7–535.9 eV) [48].

For the samples subjected to the gas oxidation (carbon dioxide or overheated water vapor) contribution of different carbon forms and the relative surface concentration of oxygen-containing moieties is comparable. However, the relative concentration of carboxyl group is relatively low in comparison to hydroxyl, ether, quinone and carbonyl groups, which is consistent with the extensive XPS studies made for the commercial carbons [38]. In the sample additionally subjected to activation by microwaves, the subsequent decrease of the carboxyl group concentration (from 7.4 to 4.4% according to the intensity of the deconvoluted peak C1s) and the increase of amounts of carbonyl or lactone groups (from 29.9 to 42% according to the intensity of deconvoluted peak O1s) is observed. Insignificant amounts of oxides, chemisorbed water and oxygen are also found in all samples, with the largest content for the material after the microwave treatment. It is worth mentioning that the concentration of respective groups detected from the XPS data is only approximate due to low extension of the applied beam (depth of several monolayers) as well as different depth of penetration of electrons for the O1s and C1s spectra resulting in varying trends for some moieties responding from both peaks.

The confirmation of a relatively low content of oxygen (oxygen-containing moieties) on the surface of the studied samples are the high values of point of zero charge determined by the potentiometric titration (Table 3). For all samples PZC is in the range of 9.5 to 11.4; however, this parameter for the sample Ac_Ar is overestimated because of a significant amount of ash. In the series of activated carbons from the pistachio shells as a precursor, the highest PZC and surface charge densities in the region of high pH values for the samples after activation using carbon dioxide and slightly lower ones for the samples after activation using water vapor and carbon dioxide were obtained. The additional activation by water vapor in microwaves or extending of the activation time, regardless of the method, causes the increase of surface oxidation and the decrease of PZC. Analyzing the results of XPS analysis and potentiometric titration one can state that the activated carbons exhibit basic characteristics of the surface that favor their application as adsorbents for removal of hydrophobic pollutants from aqueous solutions.

**Figure 9:**
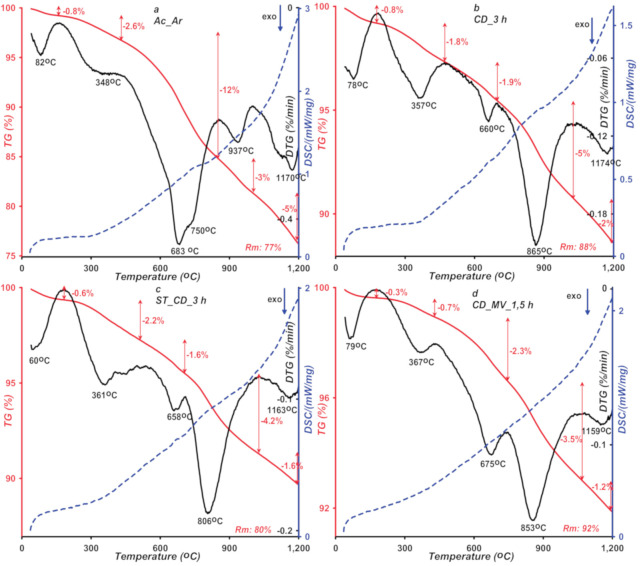
Thermogravimetric (TG), derivative thermogravimetric (DTG), and differential scanning calorimetry (DSC)curves of activated carbons (**a**–**d**) measured in the helium atmosphere.

In order to study the thermal stability of the obtained activated carbons, the thermal analysis of the samples in the helium atmosphere was carried out. Figure 9a–d shows the obtained thermogravimetric (TG), derivative thermogravimetric (DTG), and differential scanning calorimetry (DSC) curves. Based on course of the curves, four stages of the thermal degradation process can be proposed. The first stage in the range of 40 to 180 °C is related to the removal of physically adsorbed water. The mass loss at this stage is below 1% and it is the endothermic process. The subsequent stages are exothermic, and they relate to successive removal of acidic oxygen complexes or functional groups of basic character from the carbon surface.

**Figure 10:**
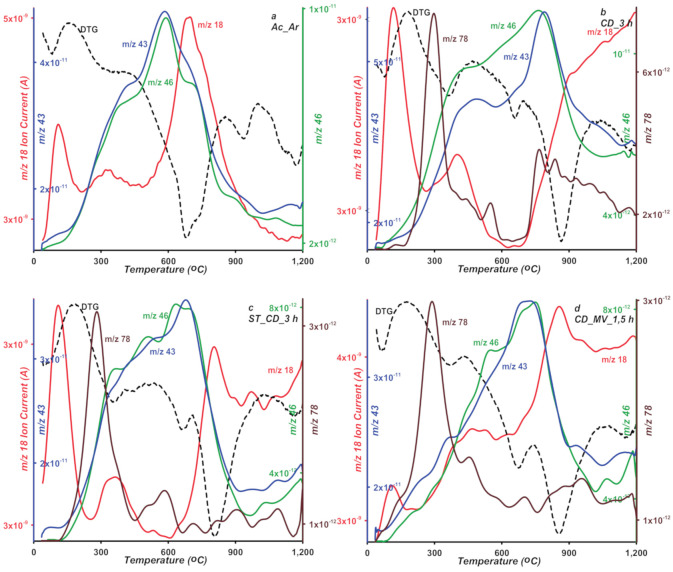
MS profiles of gaseous products of thermal degradation (water *m*/*z* 18, nitrogen dioxide *m*/*z* 46, benzene *m*/*z* 78, and acetone *m*/*z* 43) for studied samples (**a**–**d**).

**Figure 11:**
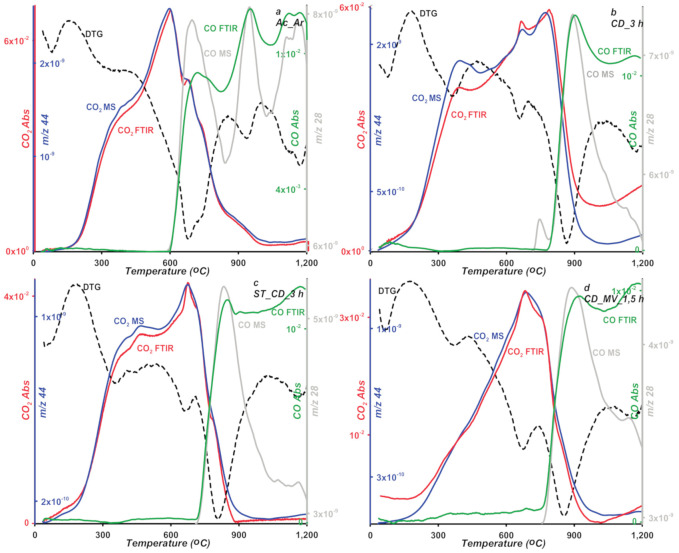
MS and FTIR profiles of gaseous products of thermal degradation (carbon dioxide *m*/*z* 44 and carbon oxide *m*/*z* 28) for studied samples (**a**–**d**).

At lower temperature range (180–450 °C), degradation of oxygen groups weakly bound to the carbons surface proceeds. The samples mass loss is within 0.7–2.6%, where the lowest and highest values correspond to the CD_MV_1,5 h and Ac_Ar samples, respectively. The largest mass loss for the Ac-Ar sample indicates greatest contribution of thermally unstable acidic groups on the carbon surface. To identify gaseous products, mass and FTIR spectrometry were applied. The results of two independent techniques are shown in Figure 10 and Figure 11. Based on the collected mass and infrared radiation spectra in the measured temperature range, the following compounds in released gaseous products were found; water (*m*/*z* 18), carbon dioxide (*m*/*z* 44), nitrogen dioxide (*m*/*z* 46), benzene (*m*/*z* 78), and acetone (*m*/*z* 43). The last two gaseous products were not detected in the Ac_Ar sample. Based on the mass and FTIR spectra, decomposing specific surface groups released during the thermal analysis process were determined. The first band on the CO_2_ profile of the tested materials at temperatures corresponding to the second peak on the DTG curve (with min. 348–367 °C) is assigned to single carboxylic groups. For the carbon ST_CD_3 h, at a slightly higher temperatures (about 440 °C) CO_2_ band corresponding to lactones or lactols, decomposition was recorded. For other samples, this band is likely overlapped by high-intensity adjacent one. In water spectra starting at 220 °C the release of water bound to oxygen complexes or from condensation process of adjacent carboxylic or phenolic species is observed. The temperature of released gaseous products consists with the literature data [49,50,51].

In the temperature range 450 to 1060 °C the oxygen species with a more complex structure and requiring a greater heat energy supply are defragmented and thermally decomposed successively. The process is accompanied by the release of volatile products mentioned in the previous stage. Additionally, starting from temperature of about 600 °C for Ac_Ar and 700 °C for the other samples, a distinguished signal of carbon monoxide appears in mass (*m*/*z* 28) and FTIR spectra. For the discussed temperature range, mass loss is within 6–15%. As in the previous temperature range, the largest mass loss for the Ac-Ar sample is observed, which indicates a greater number of acidic groups both with low thermal stability and the ones more resistant to high temperatures. Considering large mass loss of samples, distinguished peaks on the DTG curves and high intensity of signals from released gas products, the temperature range 450–1060 °C can be described as a predominant stage. Furthermore, this stage can be divided into two substages with different temperature range and course depending on a type of sample. For the Ac_Ar sample, the main stage corresponds to the DTG peak with a minimum at 700 °C and the second one corresponds to a minor peak with a minimum at 940 °C. The opposite situation is for other samples, the main peak (with min. at 800–850 °C) is preceded by the minor peak (with min. at 660–675 °C). The bands on the CO_2_ profiles with max. at 580 or 680 °C for the Ac_Ar sample and for other samples can be assigned to carboxylic anhydrides (the CO_2_ signal is accompanied by the CO one for this case) and chemically different lactones (with greater stability in comparison to the ones detected at lower temperature range). On CO profiles we can observe one or two bands originated from phenolic, hydroquinonic, carbonylic, quinonic groups, or ethers. However, a precise indication of the type of functional groups that are thermally degraded seems impossible, because temperature of the described processes depends on many variables such as: a porosity of material or a heating rate, so literature data on this issue are sometimes very divergent. In turn, on the water spectra there is a distinguished peak corresponding to temperature of the main peak on the DTG curve related with decomposition process of more stable acidic complexes. Starting from a temperature about 1060 °C degradation of alkaline moieties-pyrone structures occurs. At this stage mass loss (1.2–5%) is observed.

### 3.2. Adsorption Equilibria

**Figure 12:**
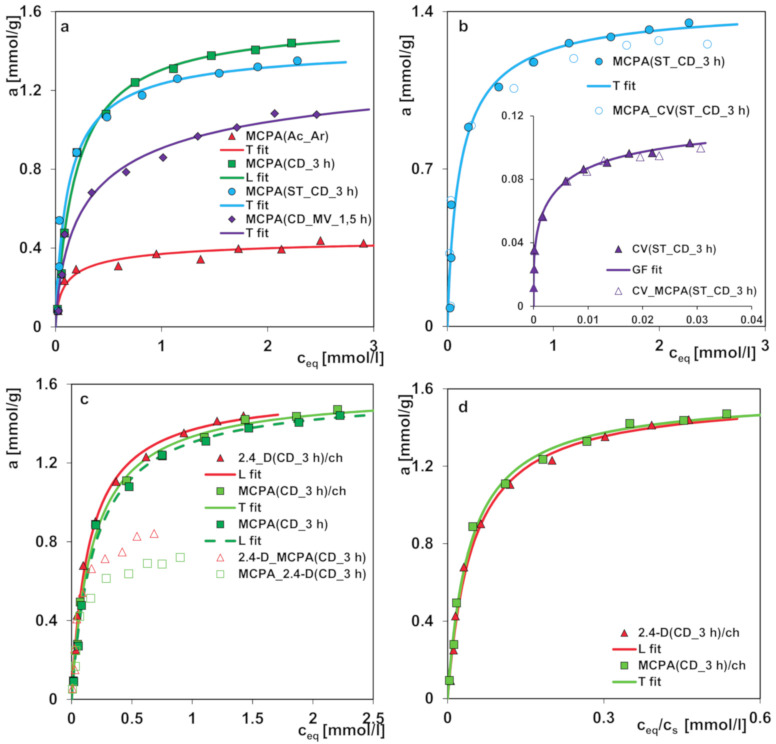
(**a**) Comparison of MCPA adsorption isotherms from the aqueous solutions on the activated carbons (**b**). Comparison of MCPA and CV isotherms from the one-component and bi-component solutions on ST_CD_3 h: the solid line for MCPA represents the chromatographic measurements and the dashed line for MCPA represents the spectrophotometric measurements (**c**). Comparison of MCPA and 2.4-D isotherms from the one-component and bi-component solutions on CD_3 h (**d**). Comparison of MCPA and 2.4-D isotherms on CD_3 h in the reduced coordinates.

The differentiation of preparation conditions, i.e., carbonization parameters and the type of agent used in the activation process, made it possible to obtain materials displaying high structural diversity. Figure 12 shows how the structural differences affect the adsorption process from the aqueous solution in both single- and multi-component systems. Regarding the choice of the presented results, the effectiveness of the adsorption process was decisive. All activated carbons from the pistachio shell series that were activated in a shorter time range exhibited worse adsorption capabilities than those obtained after the extended period of time of this stage. In Figure 12a, the adsorption isotherms for the herbicide MCPA (4-chloro-2-methyphenoxyacetic acid) on the chosen activated carbons are compared. Adsorption capacities of the adsorbents for MCPA determined by fitting the GL equation to the experimental data decrease as follows; CD_3 h > ST_CD_3 h > CD_MV_1.5 h > Ac_Ar. The adsorption capacity of CD_3 h over three times exceeds the value for Ac_Ar, whereas the changes in the adsorption capacity for the other activated carbons are not so significant. Even though CD_MV_1.5 h shows the largest specific surface area of the activated carbons, its great contribution of pores in the submicropore range makes it partly inaccessible for MCPA (*D_min_* = 0.55nm and *D_max_* = 0.95nm, where *D_min_* and *D_max_*–the distance between the remotest atoms in a molecule measured along the longitudinal and transverse axes respectively, calculated by means of Marvin 14.8.25.0 tools). Furthermore, as follows from the XPS survey and the potentiometric titration a relatively large content of surface oxygen containing moieties can result in poor effectiveness of adsorption process. A relatively large specific surface area is also characteristic for the activated carbon ST_CD_3 h, however, in this case an insignificant decrease of adsorption compared to the sample CD_3 h with a lower specific surface area is caused by remarkable contribution of mesopores to the total pore volume (68%). This indicates that the differences in adsorption of MCPA seem to be correlated rather with the pore diameters than with the specific surface areas of activated carbons (Table 3).

In the next stage of research, application of the obtained carbons as sorbents in complex systems was investigated. Natural waters contain many compounds that can compete with each other, limiting the effectiveness of adsorption technique, thus the studies on adsorption from the multicomponent solutions are of special importance. Our purpose is such a choice of sorbent with a targeted distribution of pores with the diameters enabling effective model organics adsorption from complex systems. As a model, organic pollutants different in molecular size and belonging to the group of herbicides (MCPA, 2.4-D) and dyes (crystal violet) were selected. In the adsorption studies the most effective adsorbents, ST_CD_3 h and CD_3 h, were used for the analysis of removing adsorbates of differentiated molecular sizes (MCPA and crystal violet) and the similar molecular size (MCPA and 2.4-D).

In Figure 12b, the comparison of MCPA adsorption isotherms from the one-component and bi-component solutions with crystal violet (the concentration ratio of herbicide to dye in the initial solutions was 20:1) on the activated carbon ST_CD_3 h is presented (in the inserted figure, the comparison of CV adsorption isotherms from the one-component and bi-component solutions with MCPA is shown). Only a small effect of the dye presence on the herbicide adsorption and inversely is found. This suggests that both substances are adsorbed on various adsorption sites. The large crystal violet molecules are adsorbed in the mesopores while the small MCPA ones fill mainly micropores. Thus, it can be stated that the adsorption of both compounds in the studied system is mostly independent. Undoubtedly, low CV concentration in comparison to MCPA is influential. It is highly likely that much higher concentration of dye may cause the process of blocking pores. However, the chosen methodology with spectrophotometric measurements prevents from applying a lower concentration ratio of herbicide to dye in the initial solution because high absorbance signal of dye would result in a large measurement error for herbicide due to overlapping of spectra from both adsorbates in the UV range.

In Figure 12c, the comparison of MCPA and 2.4-D adsorption isotherms from the one-component and bi-component solutions (the concentration ratio of herbicides in the initial solutions was 1:1) on the activated carbon CD_3 h is presented. Analyzing adsorption of 2.4-D and MCPA from the one-component solution slight differences between the adsorption uptake are observed, favoring the first adsorbate. The solubility of 2.4-D and MCPA is equal to 0.68 and 0.83 g/L, respectively, while the partition coefficient between the octane and water phase is *log P*: 2.46 and 2.32. Comparing the values of these parameters to the adsorption isotherms of studied systems, a close correlation between them can be clearly seen. In accordance with the general rule, effectiveness of organics adsorption depends on solubility of the compounds to a large extent. 2.4-D, as a less soluble (more hydrophobic) substance, is adsorbed more intensively on carbon by stronger hydrophobic interactions. Confirmation of the fact that solubility (hydrophobicity) of the herbicides is the main driving force in adsorption are the overlapping isotherms reduced by the solubility parameter, shown in Figure 12d. Comparing the experimental isotherms of herbicides from the one-component and binary solutions significant differences in their course are observed. Due to their relatively similar structure and physicochemical properties both compounds constitute a strong competition for each other in the adsorption process on activated carbon. However, the effect of the accompanying substance (2.4-D) on the adsorption of MCPA is stronger than that of MCPA on adsorption of 2.4-D. This may be due to the differences in the adsorption strength. For 2.4-D this parameter is higher (log K in Table 6) than for MCPA, so it favors 2.4-D in keeping already occupied activated sites or replacing them after the MCPA desorption.

Based on the values of point of zero charge for activated carbons (*pH_pzc_* = 9.5–11.4) and the experimental conditions (pH = 5.5–6), one can state that herbicides (weak acids) and dye (weak base) were mostly negatively and positively charged respectively while the charge of the activated carbons surface was positive. The acid-base properties of adsorbents and the type of adsorbate species determine possible mechanisms of the adsorption processes from the liquid phase: the dispersion interactions between π-electrons of the solute aromatic ring and the graphene layers of carbon, the solute-adsorbent electrostatic interactions or the electron donor-acceptor interactions.

For the analysis of the adsorption isotherm data for the single solute systems, the Generalized Langmuir equation (GL) was applied. The values of optimized parameters of GL equation, the regression correlations (*R*^2^) and the standard deviations (*SD*) are presented in Table 6. All the experimental data were described by the special cases of GL, i.e., Toth (T), Langmuir (L), and Generalized–Freundlich (GF) equations, as those with the minimum sum of square deviations for all experimental points *S*(*da*^2^) (general nonlinear LSQ optimization). In the case of three systems, no heterogeneity effects were found (Langmuir behavior), which can be explained by compensation of the influence of energetic nonhomogeneity and the interactions between molecules in the adsorbed phase. However, for the other adsorption systems a medium or strong energetic heterogeneity was observed (T, GF isotherms). The optimized values of adsorption capacities are the lowest for the systems: MCPA (Ac_Ar) and CV (ST_CD_3 h) which is in good agreement with the adsorption data. For the other systems the adsorption capacities are differentiated to a smaller extent. Good agreement between the experimental points and the fitted lines is visible which is also confirmed by the *SD*(*a*) and *R*^2^ values.

Due to specificity of adsorption studies of multicomponent systems, the measurements were performed using the two complementary techniques: spectrophotometric (herbicide + dye) and chromatographic (herbicide + herbicide). The compatibility of both techniques based on the course of single-solute isotherms for MCPA (the same solutions after the adsorption process were measured by the above-mentioned techniques) was confirmed and is presented in Figure 12c.

### 3.3. Adsorption Kinetics

**Figure 13:**
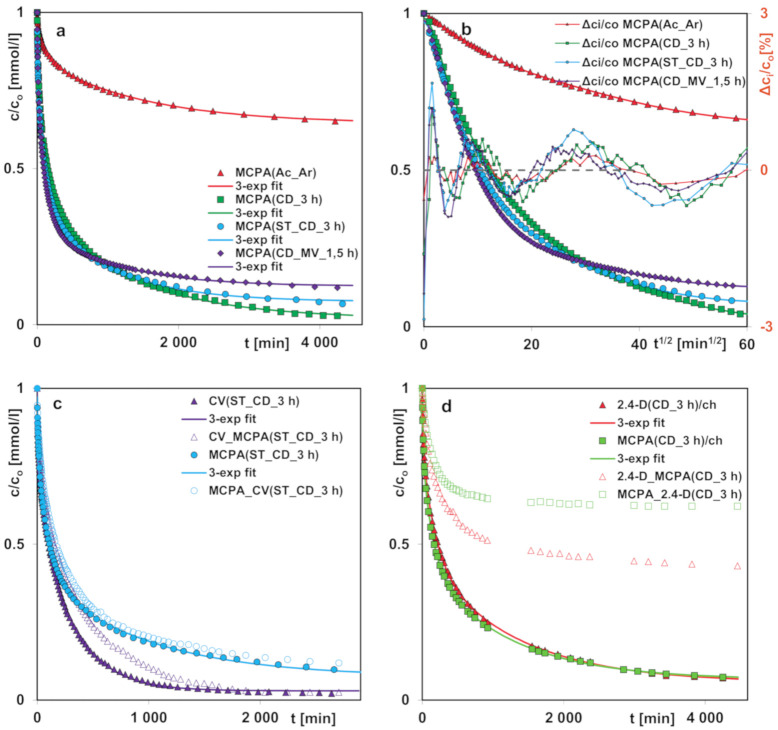
(**a**,**b**) Comparison of adsorption kinetics for MCPA on various activated carbons. (**c**). Comparison of adsorption kinetics for MCPA and CV from the one-component and bi-component solutions on ST_CD_3 h. (**d**). Comparison of adsorption kinetics for MCPA and 2.4-D from the one-component and bi-component solutions on CD_3 h. The lines correspond to the fitted multi-exponential equation.

In order to study the influence of structural properties of activated carbons on the herbicide and dye adsorption rate, the kinetic curves were measured (Figure 13a,b). From the analysis of the concentration profiles for MCPA, one can find only small differences among all studied adsorption systems. Based on the value of average adsorption half-time, the decrease of adsorption rate is observed in the following order; sample CD_MV_1.5 h > ST_CD_3 h > CD_3 h > Ac_Ar. After 10 hrs, when about 75% of adsorbate removal is obtained, the rate for the MCPA (CD_MV_1.5 h) system is slowing down, reaching the state close to equilibrium in a short period of time. Relatively fast start of the process can be related to the largest surface area of the sample and adsorption in the meso- and large micropores. Great contribution of sub-micropores, smaller than the molecular size of adsorbate limits the adsorption extent. In the case of two other samples from the pistachio series after 67 hrs change of trend, the adsorption process on the sample CD_3 h is faster than on the sample ST_CD_3 h is observed. This is associated with the differences in relative contribution of the meso- and micopores in the structure of activated carbons (large volume of the mesopores-68%, playing the role of transporting arteries in the sample ST_CD_3 h corresponds to its faster adsorption towards the herbicide and reduction of time required to reach equilibrium).

In Figure 13c the comparison of the concentration profiles for MCPA and crystal violet from the one-component and bi-component solutions (the concentration ratio of co-adsorbates in the initial solution is similar to that in the equilibrium experiment) on the activated carbon ST_CD_3 h is presented. Similarly, to the equilibrium adsorption only a slight effect of a competing substance on the herbicide adsorption rate is found. However, in the case of the crystal violet adsorption, the herbicide presence with a much higher concentration compared to that of the dye, results in a more distinct effect on adsorption kinetics. This can be explained by larger number of herbicide molecules inhibiting diffusion in the solution and adsorption space of the activated carbon. One should also pay attention to differences in the molecular size of adsorbates. The smaller herbicide molecules, in comparison to the crystal violet ones, diffuse much faster into the porous space of activated carbon and fill the active sites in the micropores. Therefore, the kinetic curves for a one-component and two-component solutions, marked in Figure 13c as CV (ST_CD_3 h) and CV (ST_CD_3 h), are very similar. The large crystal violet molecules, due to their size, diffuse and adsorb slower. Comparison of the changes in the kinetic dependencies with those in the adsorption isotherms from the one- and two-component solutions of both substances, confirms the assumption of partially independent adsorption process by filling pores of other sizes.

Figure 13d presents the comparison of MCPA and 2.4-D concentration profiles from the one-component and bi-component solutions (1:1 ratio of molar concentrations in the initial solutions) on CD_3 h. The kinetics for MCPA as a compound of smaller molecular size is slightly faster than for 2.4-D. However, in the binary systems one can see reverse behavior of both herbicides resulting from strong competition for the carbon activated sites. 2.4-D, as a compound with lower solubility, exhibits greater affinity for the hydrophobic carbon surface and negatively affects the rate of MCPA removal from the solution. The smaller MCPA molecules diffuse faster and occupy active sites in the micropores, but the presence of co-adsorbate molecules (2,4-D) in the vicinity results in successive desorption process of some MCPA molecules and return them to the bulk solution. Therefore, a significant difference in values of the relative concentration during the adsorption process for 2.4_D and MCPA in the binary solution are observed (the curves marked as 2.4-D_MCPA (CD_3 h) and MCPA_2.4-D (CD_3 h)).

The experimental kinetic profiles for the one-component adsorption systems were analyzed using the following equations; Elovich, pseudo-first-order (PFOE), pseudo-second-order (PSOE), mixed 1.2-order (MOE), as well as multi-exponential (m-exp). The nonlinear LSQ optimization of kinetic equations was applied considering the difference of experimental and optimized adsorbate concentrations, with the fitting quality estimated based on *SD*(c/c_o_) and 1-*R*^2^ values. In Table 7, the parameters (adsorption half-time, kinetic rate coefficient and relative adsorbate uptake $u_{eq}=1-\frac{c_{eq}}{c_{0}}$ calculated applying these equations are listed. The adsorption half-time (adsorption time for attaining half of uptake) for the multi-exponential equation corresponding to its various terms was obtained from the relationship: $t_{0.5}=\left(ln\;2\right)/k_{i}$, while for the total kinetics this parameter was determined numerically (bold data in Table 7). A very good correlation between the experimental data and the multi-exponential equation was obtained, whereas the other equations gave significantly worse results. Thus, in Figure 13A,B the experimental concentration profiles are presented along with the curves calculated from the m-exp equation. For better presentation of fitting quality of the m-exp equation in various ranges of measured concentrations, there are plotted in two coordinate systems the data: the relative concentration (*c/c_o_*) ~ time (*t*) and the relative concentration (*c/c_o_*) ~ square root of time (*t*^1/2^). Additionally, for all systems the deviations (Δ*c_i_/c_o_*) in the Weber-Morris linear coordinates are also shown. Generally, the greatest deviations in the initial stage of the kinetic curves are observed.

**Figure 14:**
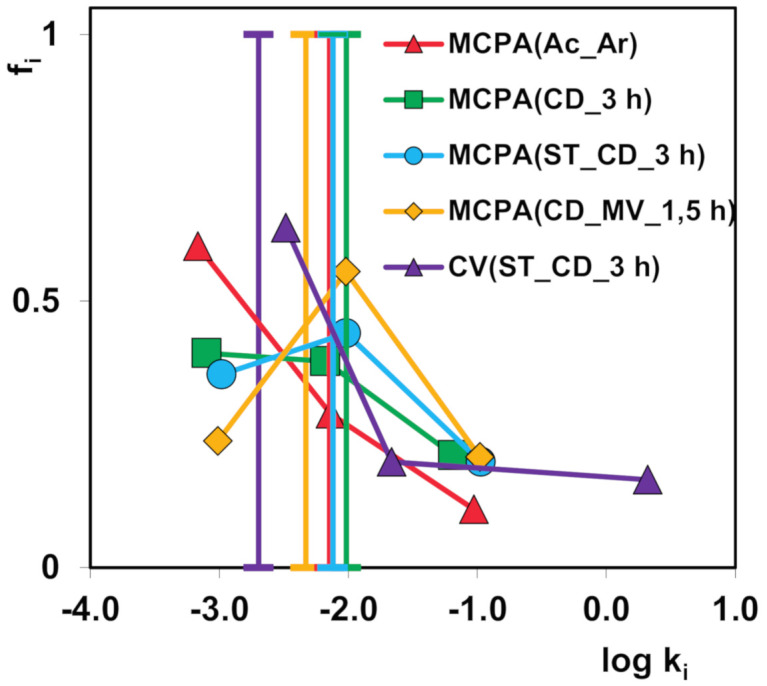
Parameter spectra for the multi-exponential equation fitted to various systems. The vertical lines correspond to the overall rate coefficients.

In Figure 14, the parameters of the multi-exponential equation as the spectrum *f_i_* vs. *log k*_i_ are presented. Each spectrum reflects the shares of terms characterized by various rate coefficients. The vertical lines correspond to the average rate coefficients calculated based on the rate coefficients for all terms of the given system. The spectra are broad because of differentiation in the rate of particular stages of the adsorption process. The most symmetric distribution of rate parameters is observed for MCPA adsorption on the samples CD_MV_1.5 h and ST_CD_3 h which indicates a small share of rate coefficients with small values. Moreover, faster kinetics on these activated carbons corresponds to the greater contribution f_i_ of the larger rate coefficients k_i_ compared to the other adsorbents (greater share *f_i_* for low *k_i_*) and larger values of their rate coefficients. A visible shift of the parameter spectrum for the CV (ST_CD_3 h) system is observed in comparison to the others.

### 3.4. Characterization of the Adsorbents after Adsorption

**Figure 15:**
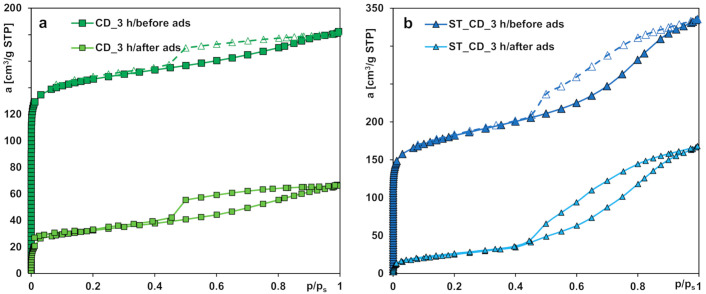
The nitrogen adsorption/desorption isotherms for the activated carbons CD_3 h (**a**) and ST_CD_3 h (**b**) before and after the herbicide adsorption.

**Figure 16:**
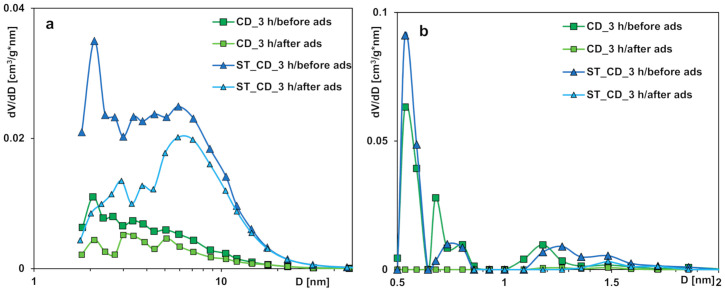
Pore size distributions of activated carbons before and after the MCPA adsorption calculated using the Barrett, Joyner, and Halenda (**a**) and the Non-Local Density Functional Theory (**b**) methods.

The comparison of the nitrogen adsorption/desorption isotherms (Figure 15), pore size distributions (Figure 16) and structural parameters (Table 8) of the activated carbons CD_3 h and ST_CD_3 h before and after MCPA adsorption, allow us to confirm that the herbicide molecules primarily fill micropores of the adsorbents. Thus, the values of specific surface area as well as the total and micropore volumes significantly decrease while the values of the median micropore width *D_mo_* increase.

## 4. Conclusions

The series of activated carbons from the strawberry seeds and pistachio shells were obtained by various methods of their chemical and physical activation. The carbon Ac_Ar, synthesized from the strawberry seeds in the process of activation with acetic acid and heating in the argon atmosphere, is characterized by the smallest development of porous structure: the smallest values of specific surface area and pore volume, and the largest ash content. The other samples were produced from the pistachio shells and pyrolysed in the same manner, changing activation time and gaseous agents or additional activation. The samples are characterized by larger values of specific surface area 555–685 m^2^/g, total pore and micropore volumes, and pH_PZC_ depending on the synthesis conditions. Generally, all samples are characterized by a relatively low content of oxygen (oxygen-containing moieties) which was confirmed by the XPS results and large values of point of zero charge determined by the potentiometric titration.

The differentiation of structural and surface properties of the obtained activated carbons influences largely the adsorption process from the aqueous solution in the single- and multi-component systems. Adsorption capacities for the selected herbicides and dye adsorbed from the one-component systems depend on the adsorbent and adsorbate structure as well as physicochemical properties. For the MCPA (CD_3 h) system the adsorption capacity exceeds these values over three times for MCPA (Ac_Ar); for the other systems, these changes are not so significant. These values are correlated with the specific surface area, micro- and mesopore volumes, pore sizes, and surface chemistry. Comparing adsorption of 2.4-D and MCPA one can find insignificant differences between adsorption uptakes; 2.4-D of lower solubility, higher hydrophobicity is adsorbed more intensely.

The effect of competing substance was analyzed for the two bi-component systems: MCPA and 2.4-D, and MCPA and crystal violet adsorbed on the most effective carbons ST_CD_3 h and CD_3 h. Comparing the adsorption isotherms from the one- and bi-component systems, a small effect of the dye presence on the MCPA adsorption was observed as a result of affinity for different adsorption sites. However, for the 2.4-D and MCPA system, a much greater influence of the competing substance was observed as a result of occupation of the same adsorption sites. The effect of 2.4-D on the MCPA adsorption is greater compared to the influence of the MCPA on 2.4-D adsorption.

A significant influence of structural and surface properties of carbons on the adsorption kinetics was also observed. Small differences among all studied one-component adsorption systems were found and the order of rate changes was established based on the values of average adsorption half-time. The differences in the herbicide kinetics were attributed to variations in the carbon pore structure: micro- and mesopore volumes, pore sizes, and adsorbate molecular structure. The analysis of concentration profiles for the bi-component systems in comparison to the one-component ones evidences an insignificant effect of the competing substance on the MCPA adsorption rate and greater effect of MCPA on the CV kinetics as a result of larger herbicide concentration. However, in the binary system: MCPA and 2.4-D one can see strong competition for the carbon activate sites. 2.4-D as a compound with lower solubility exhibits greater affinity for hydrophobic carbon surface and affects negatively the rate of MCPA removal from the solution.

## Figures and Tables

**Table 1:** Comparison of the adsorption capacities of the activated carbons with alternative non-carbonaceous adsorbents (monolayer capacity and max. sorption capacity marked with superscript 1 and 2, respectively).

Adsorbate	Activated Carbon/Precursor	Monolayer Capacity^1^/Max. Sorption Capacity^2^	Alternative Adsorbent	Monolayer Capacity^1^/Max. Sorption Capacity^2^	Ref.
copper ions	activated carbon/chickpea waste	56.2 mg/g^2^	humic acid coated sand	87.5 mg/g^2^	[1,13]
chromium (VI) ions	activated carbon/sugar beet bagasse	52.8 mg/g^2^	Polyaniline/Fe_3_O_4_ composite	174.09 mg/g^2^	[2,9]
cadmium ions	activated carbon/*Leucaena leucocephala* biomass	70.42 mg/g^2^	humic acid coated sand	18.9 mg/g^2^	[3,13]
eosin yellow dye	activated carbon/tea waste	400 mg/g^2^	chitosan/PVA composite	52.91 mg/g^1^	[4,14]
methylene blue dye	activated biochar/rice straw	90.91 mg/g^2^	lignin/chitosan composite	36.25 mg/g^2^	[5,15]
congo red dye	activated carbon/coffee waste	90.90 mg/g^1^	sand/MgFe-layered double hydroxides composite	9127.08 mg/g^2^	[6,10]
MCPA	activated carbon/wood composites	1.87 mmol/g^2^	coffee waste	0.34 g/g (1.69 mmol/g^2^)	[7,16]
methylene blue dye	activated carbon SnO_2_/corn cob	removal efficiency: 90.86%	Gum arabic-crosslinked-poly(acrylamide)/Ni(OH)_2_ /FeOOH	removal efficiency: 75%	[8,11]

**Table 2:** Physicochemical properties of the studied adsorbates.

Adsorbate Code	Chemical Formula	Molecular Weight [g/mol]	Ionization Constant pK_a_	Water Solubility [g/L]	Log P_ow_
MCPA	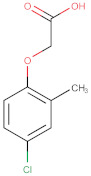	200.6	3.07 ^1^	0.825 ^2^	2.32 ^3^
2.4-D	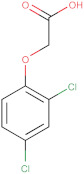	221.0	2.73 ^1^	0.680 ^2^	2.37 ^3^
CV	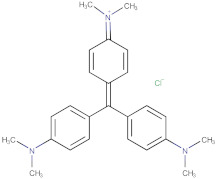	407.9	0.8 ^4^; 9.4 ^5^	17 ^6^	0.51 ^7^

^1^ Tomlin, C. The pesticide manual. Crop Protection Publications, Hampshire 1994. ^2^ Yalkowsky, S.H.; He, Y. Handbook of aqueous solubility data. CRC Press Library of Congress, Boca Raton, Floride, 2003. ^3^ Türker, L. AM1 treatment of some phenoxyacetic acid herbicides. Turkish Journal of Biology 2000, 24, 291–298. ^4^ Nalewaja, J.D.; Goss, G.R.; Tann, R.S. Pesticide Formulations and Application Systems; American Society for Testing and Materials: West Conshohocken, 1997. ^5^ Crini, G.; Badot, P.-M. Sorption Processes and Pollution: Conventional and Non-Conventional Sorbents; Besançon Presses universitaires de Franche-Comté, 2010. ^6^ Suvarna, S.K.; Layton, C.; Bancrof, J.D. Bancroft’s Theory and Practice of Histological Techniques, Churchill Livingstone, 2018. ^7^ Toxicology Data Network, https://toxnet.nlm.nih.gov/.

**Table 3:** The values of parameters characterizing the porous structure of adsorbents.

Activated Carbon	*S_BET_* [m^2^/g]	*S_ext_* [m^2^/g]	*V_t_* [cm^3^/g]	*V_mic_* (T-Plot) [cm^3^/g]	*D_mo_* (H-K) [nm]	*pH_pzc_*
Ac_Ar	243	84	0.12	0.07	1.05	9.7
CD_1 h	599	114	0.27	0.20	0.50	11.4
CD_3 h	556	116	0.28	0.18	0.55	11.1
ST_CD_1 h	587	166	0.37	0.17	0.69	11
ST_CD_3 h	669	247	0.52	0.17	1.50	10.4
CD_MV_1 h	646	164	0.30	0.19	0.55	9.9
CD_MV_1,5 h	685	104	0.35	0.24	0.59	9.5

**Table 4:** The microstructural properties of activated carbons determined by Porod evaluation.

Activated Carbon	*k_p_*	Porod Approximation	
		$\frac{S}{V}$, [Å^−1^]	S_SAXS_, [m^2^/g]
CD_1 h	0.407	0.1302	620
CD_3 h	0.760	0.1344	592
ST_CD_1 h	0.939	0.1134	540
ST_CD_3 h	1.276	0.1155	550
CD_MV_1 h	0.525	0.1562	743
CD_MV_1.5 h	0.807	0.1621	771

**Table 5:** The contribution of carbon, oxygen species, and atomic percentage of elements on the surface of carbons tested from the X-ray photoelectron spectroscopy (XPS) analysis.

B.E. (eV)	Group	Ac_Ar	CD_3 h	ST_CD_3 h	CD_MV_1.5 h
**C1s (%)**					
284.6–284.7	C-C, C-H/sp^3^	27.4	30.9	31.0	26.3
284.2–284.3	C=C/sp^2^	30.9	13.2	13.5	14.4
284.9–285	C-C high	16.4	28.2	28.6	24.7
285.5–285.7	C-O-C, C-OH	13.1	10.4	12.2	15.6
286.3–286.7	C=O	12.3	9.9	8.8	13.4
288–288.8	COO	-	7.4	5.9	4.4
290.2	OCOO	-	-	-	1.3
**O1s(%)**					
529.9–530.4	O (oxides)	4.2	5.4	1.3	8.7
531.3–532	O=C	23.3	29.9	30.4	42.0
532.3–533	C-OH, C-O-C/aliph	37.6	34.2	33.7	20.7
533.1–533.8	C-OH, C-O-C/arom	31.5	26.9	30	21.9
534.7–535.9	H_2_O, O_2_/ads	3.4	3.7	4.6	6.7
**Element (% at)**					
carbon		86.8	94.7	94.4	94.1
oxygen		9.5	5.4	5.6	5.9
nitrogen		2.5	-	-	-
phosphorous		0.8	-	-	-
silicon		0.4	-	-	-

**Table 6:** Parameters of the Generalized Langmuir equation for the organics adsorption on the activated carbons.

System	Isotherm	*a*_m_	*m*	*n*	Log *K*	*R* ^2^	*SD*(*a*)
MCPA (Ac_Ar)	T	0.47	1	0.57	1.45	0.935	0.031
MCPA (CD_3 h)	L	1.56	1	1	0.72	0.992	0.046
MCPA (ST_CD_3 h)	T	1.44	1	0.85	1.01	0.961	0.103
MCPA (CD_MV_1.5 h)	T	1.43	1	0.58	0.91	0.976	0.062
2.4-D (CD_3 h)/ch	L	1.57	1	1	0.83	0.992	0.050
MCPA (CD_3 h)/ch	T/L	1.56	1	0.99	0.76	0.994	0.044
CV (ST_CD_3 h)	GF	0.12	0.26	1	1.66	0.993	0.003

**Table 7:** Comparison of the parameters of various kinetic equations for the studied systems.

Kinetic System	Fit	*i*	*f_i_*	log k*	*t_0.5_* [min]	*u_eq_*	*SD(c/c_o_)*	*1-R* ^2^
MCPA (Ac_Ar)	Elovich	-	-	-	-	-	0.35%	1.1 × 10^−3^
	FOE	2	0	−2.73	371	0.31	1.91%	3.4 × 10^−2^
	SOE	2	1	−2.59	389	0.35	1.22%	1.4 × 10^−2^
	MOE	2	0.99	−7	389	0.35	1.24%	1.4 × 10^−2^
	m-exp	1	0.11	−1.02	7	0.36	0.21%	3.4 × 10^−4^
		2	0.29	−2.14	95			
		3	0.60	−3.16	1008 (av.342)			
MCPA (CD_3 h)	Elovich	-	-	-	-	-	1.59%	3.0 × 10^−3^
	FOE	2	0	−2.45	195	0.89	5.58%	4 × 10^−2^
	SOE	2	1	−2.25	179	0.97	2.97%	1.1 × 10^−2^
	MOE	2	0.99	−5.218	178	0.96	2.99%	1.1 × 10^−2^
	m-exp	1	0.21	−1.19	11	0.98	0.39%	1.9 × 10^−4^
		2	0.39	−2.18	104			
		3	0.40	−3.10	867 (av. 148)			
MCPA (ST_CD_3 h)	Elovich	-	-	-	-	-	2.48%	8.0 × 10^−3^
	FOE	2	0	−2.27	128	0.85	5.07%	3.5 × 10^−2^
	SOE	2	1	−2.07	117	0.91	2.59%	8.9 × 10^−3^
	MOE	2	0.99	−5.00	117	0.91	2.61%	8.9 × 10^−3^
	m-exp	1	0.20	−0.97	6	0.93	0.49%	3.2 × 10^−4^
		2	0.44	−2.01	72			
		3	0.36	−2.98	664 (av. 97)			
MCPA (CD_MV_1,5 h)	Elovich	-	-	-	-	-	4.02%	3.1 × 10^−2^
	FOE	2	0	−2.10	92	0.81	3.97%	3 × 10^−2^
	SOE	2	1	−1.90	79	0.87	1.35%	3.4 × 10^−3^
	MOE	2	0.99	−4.90	78	0.87	1.36%	3.4 × 10^−3^
	m-exp	1	0.21	−0.98	7	0.88	0.50%	4.3 × 10^−4^
		2	0.55	−2.02	72			
		3	0.24	−3.01	709 (av. 72)			
2.4-D (CD_3 h)/ch	Elovich	-	-	-	-	-	1.49%	2.7 × 10^−3^
	FOE	2	0	−2.58	262	0.87	4.93%	3.2 × 10^−2^
	SOE	2	1	−2.39	247	0.95	2.82%	1.0 × 10^−2^
	MOE	2	0.99	−4.67	247	0.95	2.86%	1.0 × 10^−2^
	m-exp	1	0.20	−1.17	10	0.95	0.36%	1.4 × 10^−4^
		2	0.37	−2.26	126			
		3	0.43	−3.11	884 (av. 192)			
MCPA (CD_3 h)/ch	Elovich	-	-	-	-	-	1.73%	3.9 × 10^−3^
	FOE	2	0	−2.50	218	0.87	5.33%	4.0 × 10^−2^
	SOE	2	1	−2.27	188	0.93	2.94%	1.1 × 10^−2^
	MOE	2	0.99	−5.29	210	0.96	3.32%	1.3 × 10^−2^
	m-exp	1	0.21	−1.15	10	0.93	0.32%	1.5 × 10^−4^
		2	0.38	−2.15	98			
		3	0.41	−3.06	804 (av. 144)			
CV (ST_CD_3 h)	Elovich	-	-	-	-	-	3.96%	2.0 × 10^−2^
	FOE	2	0	−2.33	149	0.95	2.95%	1.1 × 10^−2^
	SOE	2	1	−2.09	122	1	2.63%	9.6 × 10^−3^
	MOE	2	0.80	−2.87	136	0.99	1.90%	4.6 × 10^−3^
	m-exp	1	0.16	0.32	0.33	0.97	0.42%	2.1 × 10^−4^
		2	0.20	−1.66	32			
		3	0.64	−2.48	211 (av. 91)			

k*: k_1_-FOE and MOE; k_2_-SOE; k_avg_(t),=ln2/t_05_ -m-exp.

**Table 8:** Comparison of structural parameters of activated carbons before and after the MCPA adsorption.

ActivatedCarbon	*S_BET_* [m^2^/g]	*S_ext_* [m^2^/g]	*V_t_* [cm^3^/g]	*V_mic_* (t-plot) [cm^3^/g]	*D_mo_* (H-K) [nm]
CD_3 h/before ads.	556	116	0.28	0.18	0.55
CD_3 h/after ads.	115	67	0.12	0.02	4.40
ST_CD_3 h/before ads.	669	247	0.52	0.17	1.50
ST_CD_3 h/after ads.	230	209	0.25	-	8.49
